# Polyunsaturated Fatty Acid Supplementation in Multiple Sclerosis Management: A Scoping Review

**DOI:** 10.3390/nu18172915

**Published:** 2026-09-05

**Authors:** Rita Oliveira, Carla M. Lopes, Cláudia Silva, Rita S. Guerra

**Affiliations:** 1 Faculty of Health Sciences, Fernando Pessoa University, 4200-150 Porto, Portugal; csilva@ufp.edu.pt (C.S.); ritaguerra@ufp.edu.pt (R.S.G.); 2 RISE-Health, Fernando Pessoa Teaching and Culture Foundation, Fernando Pessoa University, 4200-150 Porto, Portugal; 3 UCIBIO—Applied Molecular Biosciences Unit, MedTech, Laboratory of Pharmaceutical Technology, Faculty of Pharmacy, University of Porto, 4050-313 Porto, Portugal; 4 Associate Laboratory i4HB, Institute for Health and Bioeconomy, Faculty of Pharmacy, University of Porto, 4050-313 Porto, Portugal; 5 LAETA—Associate Laboratory for Energy, Transports and Aerospace, INEGI—Institute of Science and Innovation in Mechanical and Industrial Engineering, 4200-465 Porto, Portugal

**Keywords:** multiple sclerosis, polyunsaturated fatty acids, omega-3, omega-6, fish oil, supplementation, scoping review

## Abstract

Polyunsaturated fatty acids (PUFA) have long been investigated as potential adjunctive therapies for multiple sclerosis because of their immunomodulatory, anti-inflammatory, and neuroprotective properties. However, the available evidence remains heterogeneous and has not been comprehensively mapped. This scoping review aimed to characterize the evidence on PUFA supplementation, identify research trends and knowledge gaps, and contextualize findings from primary studies alongside systematic reviews. This scoping review followed the Joanna Briggs Institute methodology and the PRISMA-ScR guidelines. MEDLINE (via PubMed), Scopus, Web of Science, ScienceDirect, Cochrane CENTRAL, ClinicalTrials.gov, WHO ICTRP, and EU CTR/EudraCT were searched up to June 2026. Eligible evidence included randomized and non-randomized intervention studies, longitudinal cohort studies, systematic reviews, and clinical trial registry records evaluating omega-3, omega-6, or combined PUFA supplementation in persons with multiple sclerosis. Data were descriptively charted and narratively synthesized. Thirty reports representing 24 independent evidence sources (18 primary studies and 6 systematic reviews) were included. Research evolved from early omega-6 interventions, which demonstrated biological activity but limited clinical benefit, to omega-3 supplementation, where mechanistic evidence expanded despite inconsistent clinical outcomes. More recent studies investigated combined PUFA formulations, reporting the most promising findings for relapse-related outcomes and inflammatory biomarkers, while evidence remains limited by a small number of studies and heterogeneous interventions. Systematic reviews consistently interpreted the evidence more cautiously than individual primary studies. PUFA research in multiple sclerosis has progressed from exploratory studies to more sophisticated intervention strategies integrating clinical and mechanistic outcomes. Although PUFAs show biological activity and favorable safety profiles, consistent evidence of clinically meaningful disease modification remains limited. This evidence map highlights methodological advances, persistent heterogeneity, and priorities for future well-designed randomized controlled trials.

## 1. Introduction

Multiple sclerosis (MS) is a chronic, inflammatory, immune-mediated neurodegenerative disease, characterized by demyelination and neurodegeneration of the central nervous system [1]. The reduction and progressive loss of the myelin sheath cause a variety of clinical manifestations depending on the system involved [2], such as cognitive impairment, tremor, clumsiness and poor balance, impaired swallowing and speech, unilateral painful loss of vision, weakness, painful spasms, bladder dysfunction, or pain.

MS manifests in three main clinical presentations: Relapsing–Remitting MS (RRMS), which is the most common phenotype, representing approximately 85–90% of newly diagnosed cases, Secondary Progressive MS (SPMS), and Primary Progressive MS (PPMS), which affects 10–15% of patients [3].

According to the Atlas of MS [4], there are 2.9 million people around the world with MS. Even though global prevalence has increased, it remains higher in high-income countries (35%), and among women, who account for 69% of cases worldwide.

Although MS remains incurable [5], the introduction of disease-modifying therapies (DMTs) has substantially improved disease management by reducing relapse frequency and delaying disability progression. Current DMTs encompass several therapeutic classes with distinct mechanisms of action, including interferons, glatiramer acetate, teriflunomide, fumarates, sphingosine-1-phosphate receptor modulators such as fingolimod and siponimod, cladribine, monoclonal antibodies targeting immune cell trafficking or depletion, and Bruton tyrosine kinase inhibitors. Nevertheless, therapeutic response remains heterogeneous, progressive neurodegeneration is only partially controlled, and no treatment is currently curative, sustaining interest in complementary strategies capable of modulating inflammation, oxidative stress, and neuroprotection [6,7]. Therefore, it is not surprising that many MS patients use complementary and alternative therapies as part of their treatment [8]. Given the complexity and multifactorial nature of MS pathogenesis, which includes environmental factors, namely nutritional factors such as low vitamin D levels and, in particular, reduced polyunsaturated fatty acid (PUFA) intake, nutritional intervention represents one of the potential therapeutic strategies [9,10].

Interest in dietary lipids emerged following the ecological observations of Swank and colleagues in 1952, who reported lower MS prevalence in Norwegian coastal populations consuming fish-rich diets than in inland communities with higher saturated fat intake, suggesting a potential protective role for dietary PUFAs [11]. After this publication, investigation regarding PUFAs and MS gained ground.

PUFAs are grouped into two major families—omega-3 and omega-6—based on the position of the first double bond from the methyl end of the carbon chain. Linoleic acid (LA) is the main dietary omega-6 fatty acid, while α-linolenic acid (ALA) is the main dietary omega-3 form. Within the omega-3 family, eicosapentaenoic acid (EPA) and docosahexaenoic acid (DHA) are also represented in the diet, and within the omega-6 family, arachidonic acid (AA) is a key representative. It is worth mentioning that although EPA and DHA can be synthesized from ALA, conversion efficiency is limited, making dietary intake their principal source [12].

The biological rationale for PUFA supplementation in MS is supported by the diverse physiological functions of these fatty acids within the central nervous system. PUFAs are essential structural components of neuronal and myelin membranes, maintaining membrane fluidity and acting as precursors of numerous bioactive lipid mediators involved in cellular signaling and tissue homeostasis [13]. Beyond their structural role, omega-3 fatty acids exert important immunomodulatory effects by regulating inflammatory signaling pathways, modulating cytokine production, and promoting a shift towards a less pro-inflammatory immune phenotype, whereas omega-6 fatty acids participate in membrane homeostasis and lipid mediator synthesis [13,14]. Collectively, these biological properties provide a mechanistic rationale for investigating PUFA supplementation as a complementary strategy in MS. Whereas clinical evidence remains heterogeneous, these mechanisms have sustained interest in evaluating PUFA supplementation as an adjunct to conventional DMTs [15].

Recent evidence synthesis has strengthened the rationale for investigating PUFAs in immune-mediated diseases. An umbrella review combining systematic reviews with Mendelian randomization analyses found the most consistent evidence for omega-3 fatty acids in rheumatoid arthritis and systemic lupus erythematosus, while evidence for MS and several other autoimmune diseases remained limited or inconsistent despite signals of potential benefit. Although findings from other autoimmune diseases cannot be directly extrapolated to MS because of differences in disease-specific pathophysiology and clinical outcomes, they provide biological plausibility for investigating the immunomodulatory potential of PUFAs. The comparatively limited and inconsistent evidence in MS therefore reinforces the need for disease-specific evidence synthesis [16].

The limited availability of PUFAs in the diet, combined with Western diets’ high omega-6 intake, hinders achieving the recommended 5:1 ratio [17,18]. Although adherence to a healthy dietary pattern is also recommended for people with MS, dietary intake alone cannot reliably achieve the standardized PUFA exposure required to investigate biological effects relevant to MS pathophysiology. The PUFA composition of natural foods varies according to species, cultivation conditions, processing, and preparation, making it difficult to ensure consistent daily intakes of individual fatty acids or predefined omega-6/omega-3 ratios [12]. Consequently, studies evaluating standardized PUFA supplementation address a fundamentally different research question than studies investigating habitual dietary intake or dietary patterns. Nutraceutical formulations provide precisely defined doses and compositions, allowing for consistent exposure across participants and facilitating the evaluation of dose–response relationships and treatment efficacy. This standardization is particularly important because several clinical trials reporting favorable outcomes have employed PUFA doses that would be difficult to obtain consistently through usual dietary intake alone [19]. Furthermore, therapeutic response is determined by both the administered dose and the pharmaceutical formulation. Unlike dietary sources, where digestion and absorption are influenced by meal composition and the food matrix, nutraceutical formulations can be specifically designed to enhance PUFA bioavailability. Technologies that enhance lipid emulsification and absorption have been shown to increase the systemic availability of long-chain omega-3 fatty acids [20], and when combined with antioxidant components, these formulations protect unsaturated lipids from peroxidation [21,22,23], improving absorption, membrane incorporation, and biological activity, while reducing inter-individual variability in PUFA exposure.

Taken together, these considerations indicate that the current literature is characterized not only by heterogeneous clinical findings but also by considerable diversity in PUFA formulations, doses, and intervention strategies. Despite the widespread interest in PUFA supplementation as a complementary strategy for patients with MS, the available evidence remains inconsistent and formal clinical guidelines are lacking. This knowledge gap highlights the need to systematically characterize the scope, nature, and development of the available evidence.

This scoping review aimed to systematically map and characterize the available evidence on omega-3, omega-6, and combined PUFA supplementation in people with MS. The review focuses specifically on supplemental PUFAs rather than dietary intake, as standardized formulations provide controlled doses and compositions that allow a more reliable assessment of biological and clinical effects than a typical diet alone. Although PUFA supplementation has been investigated for several decades, the available evidence remains highly heterogeneous in terms of formulations, dosages, intervention duration, comparator groups, clinical and biological outcomes, and study designs. Given this heterogeneity, a scoping review represents the most appropriate methodological approach to comprehensively map the existing evidence, characterize the evolution of the field, identify knowledge gaps, and inform priorities for future mechanistic research and well-designed clinical trials.

## 2. Materials and Methods

### 2.1. Protocol and Registration

This scoping review was conducted in accordance with the Joanna Briggs Institute (JBI) methodology for scoping reviews [24] and reported in accordance with the Preferred Reporting Items for Systematic Reviews and Meta-Analyses extension for Scoping Reviews (PRISMA-ScR) guidelines [25]. The review protocol was prospectively registered in the Open Science Framework (OSF) (https://doi.org/10.17605/OSF.IO/TNBMF, accessed on 25 August 2026).

### 2.2. Eligibility Criteria

Eligibility criteria were established a priori according to the Participants–Concept–Context (PCC) framework recommended by the JBI for scoping reviews [24].

The participants comprised humans diagnosed with MS (all clinical phenotypes), clinically isolated syndrome (CIS), or a first demyelinating event of the central nervous system. There were no restrictions on age, gender, disease subtype, disease duration, geographical location, or clinical setting.

The concept was supplementation with PUFAs administered in clearly defined galenic dosage forms, including omega-3, omega-6, and combined PUFA formulations. Multicomponent interventions were considered eligible only if the PUFA component was clearly defined, and its contribution could be reasonably interpreted independently of the accompanying intervention. Studies investigating dietary patterns, nutritional counselling, or consumption of PUFA-rich foods were excluded because the effects of supplementation could not be distinguished from those of the overall diet.

The context was unrestricted, with no limitations regarding geographical location or clinical setting.

Eligible sources of evidence comprised randomized and non-randomized intervention studies, longitudinal cohort studies, systematic reviews, and clinical trial registry records. Narrative reviews, editorials, conference abstracts, case reports, cross-sectional and case–control studies were excluded. Only registry records that contained publicly available extractable results or linked publications with extractable data were considered eligible. No lower publication-date limit was applied, and no language restrictions were imposed.

Supplementary Table S1 provides detailed operational eligibility criteria.

### 2.3. Information Sources

Electronic searches were conducted in MEDLINE (via PubMed), Scopus, Web of Science Core Collection, ScienceDirect, and the Cochrane Central Register of Controlled Trials (CENTRAL). Clinical trial registries, including ClinicalTrials.gov, the World Health Organization International Clinical Trials Registry Platform (WHO ICTRP), and the European Union Clinical Trials Register (EU CTR/EudraCT), were also searched to identify eligible registered studies and linked publications. To maximize retrieval, the reference lists of systematic reviews identified during the electronic search were manually screened before full-text eligibility assessment to identify potentially eligible primary studies that had not been retrieved by the database searches. All database and trial registry searches were carried out on 12–13 June 2026, covering the available literature up to the date of the final search.

### 2.4. Search Strategy

The search strategy was developed to retrieve both primary studies and systematic reviews relevant to the review objectives, following the JBI approach. An initial exploratory search of MEDLINE (via PubMed) and Scopus was undertaken to identify relevant search terms. These terms were subsequently used to develop comprehensive database-specific search strategies that were adapted to the syntax and field tags of each information source. Search strategies combined terms related to (i) multiple sclerosis and related demyelinating disorders and (ii) polyunsaturated fatty acid supplementation, including omega-3, omega-6, and related formulations. No search limits or methodological filters were applied to maximize the sensitivity of the search strategy. Supplementary Table S2 contains complete search strategies for all information sources.

### 2.5. Selection of Sources of Evidence

The initial title and abstract screening was conducted using a large language model (LLM)-assisted workflow (OpenAI ChatGPT, GPT-5.5 Thinking) based on a standardized screening prompt developed specifically for this review. Using the predefined operational eligibility criteria, the LLM identified potential bibliographic duplicate records and classified each remaining record as Include, Uncertain, or Exclude, providing a brief justification for each classification based solely on the available title, abstract, and bibliographic metadata. A conservative screening approach was adopted, in which records with insufficient information to determine eligibility were classified as Uncertain rather than excluded. A human reviewer manually verified all bibliographic duplicates and records classified as Exclude by the LLM to confirm consistency with the predefined eligibility criteria. Records classified as Include or Uncertain were transferred to Rayyan for human assessment. Because human verification encompassed the complete set of LLM-classified exclusions, no separate independent human sample was screened to estimate missed-study risk. This workflow was designed to prioritize sensitivity during the initial screening stage while ensuring human oversight of all exclusion decisions. Supplementary File S1 provides the complete LLM prompting framework and screening workflow.

Full-text eligibility assessment was then conducted independently by two reviewers using the Rayyan systematic review platform (Rayyan Systems, Inc., Cambridge, MA, USA; free version accessed in June 2026). Disagreements were resolved through discussion and, when necessary, consultation with a third reviewer.

### 2.6. Data Charting Process and Data Items

Data were charted using structured forms developed a priori to ensure consistent charting across all evidence sources included. For primary studies, core variables extracted included the evidence source, study design, population, PUFA intervention or exposure, comparator, intervention duration, and reported clinical and biological outcomes. For systematic reviews, extracted variables included the evidence source, review type, number of studies included, PUFA interventions covered, and principal outcomes. Supplementary Table S3 presents the complete data charting forms. To improve the efficiency of data charting, Google’s NotebookLM was used as a closed-source, context-constrained retrieval assistant operating exclusively on the full-text articles uploaded for this review. NotebookLM was used to locate relevant information within the included reports and to facilitate the retrieval of additional contextual information during data charting and narrative synthesis. All information retrieved using NotebookLM was manually verified against the original publications before being incorporated into the review. Data charting was performed by one reviewer. Uncertain or ambiguous information was discussed with a second reviewer, and all charted data were verified against the original publications prior to inclusion in the review. Supplementary File S1 provides the complete NotebookLM prompting framework and data charting workflow.

### 2.7. Evidence Characterization

In accordance with the objectives of a scoping review, no formal critical appraisal or risk-of-bias assessment was undertaken. Thus, no validated appraisal tools were applied, no quality scores were assigned, and study findings were not weighted based on methodological quality. Instead, a structured evidence characterization was performed for all included evidence sources to support descriptive mapping and contextual interpretation of the evidence. The characterization included study features that were considered relevant to the interpretation, reproducibility, and contextualization of the evidence, such as study design, completeness of PUFA intervention characterization, dose and intervention duration, presence of a comparator group, outcome domains assessed, and PUFA-specific interpretability. These characteristics were summarized descriptively without assigning methodological ratings or levels of evidence, and they were not intended to be used to assess methodological quality.

For included systematic reviews, additional methodological characteristics were charted to contextualize the secondary evidence, including the search period covered, databases searched, critical appraisal or risk-of-bias tools applied, and any reported assessment of the certainty or overall quality of the evidence.

### 2.8. Data Synthesis

The objectives of a scoping review were followed in the descriptive synthesis of extracted data. Study characteristics were summarized in structured evidence tables separately for primary studies and systematic reviews. The findings were subsequently narratively synthesized according to PUFA intervention category (omega-6, omega-3, and combined PUFA formulations). Primary studies and systematic reviews were synthesized separately, with systematic reviews used to contextualize the primary evidence, identify areas of agreement or divergence, and provide an overview of the broader evidence base rather than duplicate individual study findings. Evidence characterization provided additional context regarding the methodological and reporting characteristics of the included evidence sources without requiring formal methodological appraisal or informing study weighting. When two or more reports referred to the same underlying study or participant cohort, they were identified and grouped into a publication family and treated as a single independent evidence source. Data were synthesized at the evidence source level, incorporating information from linked reports to maximize completeness while avoiding duplication of evidence.

## 3. Results

### 3.1. Search Results

**Figure 1:**
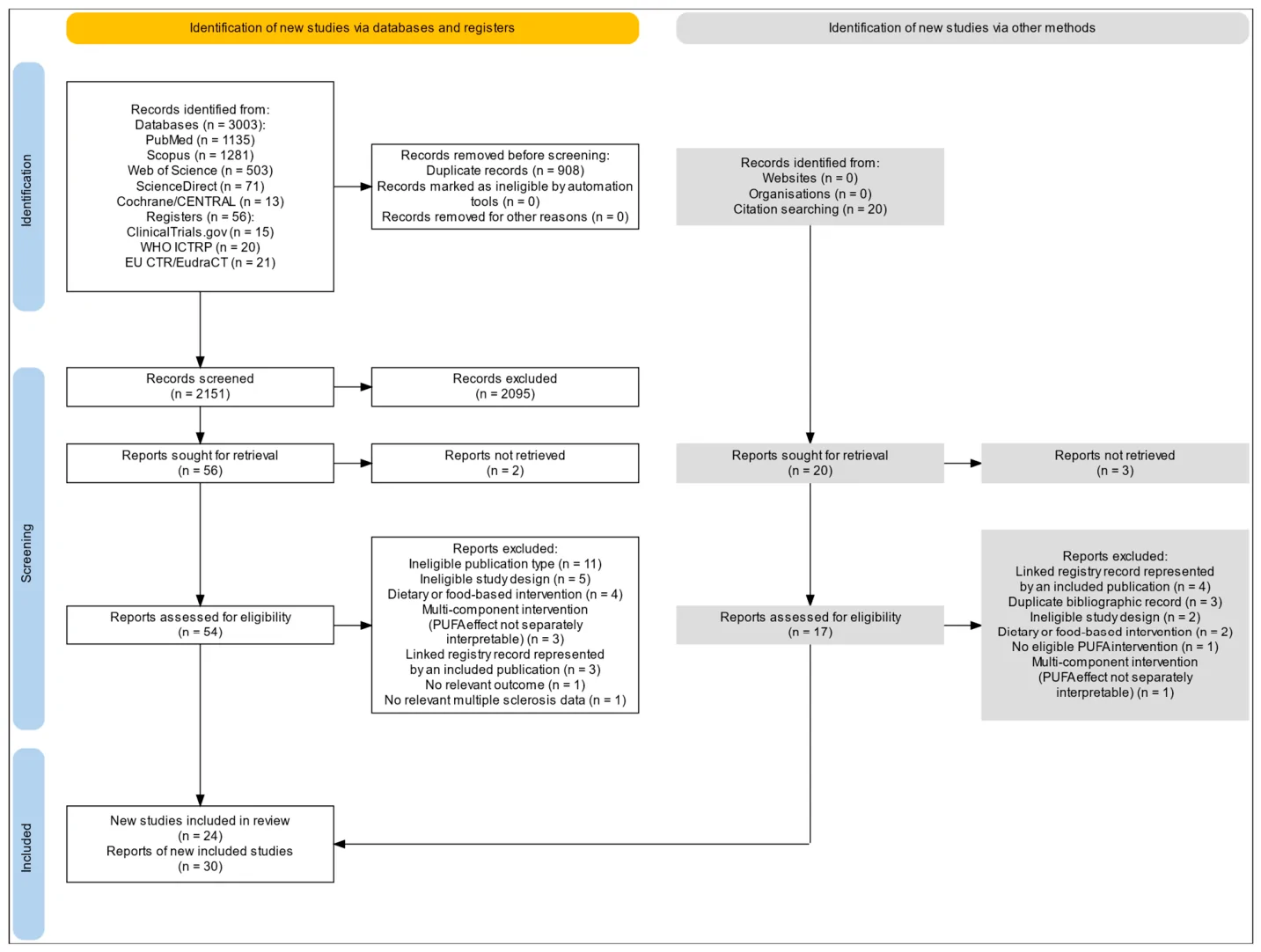
PRISMA 2020 flow diagram [26].

The database search identified 3003 records across PubMed (*n* = 1135), Scopus (*n* = 1281), Web of Science (*n* = 503), ScienceDirect (*n* = 71), and Cochrane/CENTRAL (*n* = 13). A further 56 records were identified through clinical trial registers: ClinicalTrials.gov (*n* = 15), WHO ICTRP (*n* = 20), and EU CTR/EudraCT (*n* = 21). Manual screening of the reference lists of relevant systematic reviews identified 20 additional records. Following the removal of 908 duplicates, 2151 unique records were screened for title and abstract with 2095 being excluded. At the initial LLM-assisted screening stage, 40 records were classified as Include, 15 as Uncertain, and 1960 as Exclude. Following human verification of the LLM-classified exclusions, one record was reclassified for full-text assessment (1/1960; 0.05%). Seventy-six reports were sought for retrieval, five of which were not retrievable, leaving 71 reports to be assessed for eligibility. Thirty-eight reports were excluded following full-text eligibility assessment, while three duplicate bibliographic records identified at this stage were removed. Supplementary Table S4 lists the excluded reports and explains the reasons for exclusion. Thirty reports met the eligibility criteria and were included in the review, corresponding to 24 independent evidence sources, comprising 18 primary studies and six systematic reviews (Figure 1). Two publication families were identified, corresponding to the Ramirez-Ramirez and Rezapour-Firouzi studies.

The 18 primary studies were published between 1977 and 2026 and were conducted in the United Kingdom (5), Iran (4), Cyprus (3), the United States (2), Mexico (1), Norway (1), Italy (1), and Iraq (1). The primary studies investigated omega-6, omega-3, and combined PUFA interventions across MS phenotypes. The six systematic reviews, published between 2017 and 2022, are presented independently of the primary studies.

The included primary studies encompassed all major multiple sclerosis phenotypes, including RRMS, SPMS, PPMS, and chronic progressive or mixed MS populations. No eligible studies specifically investigated PUFA supplementation in participants with CIS or a first demyelinating event, although participants with CIS were included within the mixed population of Golabi et al. [27].

### 3.2. Characteristics of Included Primary Studies

#### 3.2.1. Omega-6 Studies

Omega-6 supplementation research comprised four primary studies published between 1977 and 1983 (Table 1). The research group led by Bates et al. conducted two randomized clinical trials (RCT) [28,29], whereas Field et al. conducted two observational reports that explored biological responses to long-term supplementation [30,31]. Sample size ranged from 28 to 152 participants, with intervention durations varying from 6 months to 3 years. The included studies enrolled patients with different MS phenotypes, including chronic progressive, RRMS, and established ambulatory disease. In the RCT, baseline demographic and clinical characteristics were generally comparable between treatment groups.

All interventions used LA-rich formulations based on Naudicelle oil, which were either administered as capsules or incorporated into spreads. Daily LA doses varied substantially across studies, ranging from approximately 2.6 g to 23 g. Two randomized trials also included supplementation with small amounts of γ-linolenic acid, whereas comparator groups generally received oleic acid. In observational studies, adherence was primarily monitored through serial biochemical assessments of erythrocyte membrane responses, whereas the randomized trials additionally evaluated changes in serum fatty acid composition. Omega-6 supplementation was generally well tolerated, with no serious treatment-related adverse events reported. RCT’s safety assessments included routine laboratory monitoring.

The RCT primarily evaluated relapse frequency, severity and duration, disability progression assessed using the Kurtzke Disability Status Scale, and standardized neurological examinations. In contrast, observational studies focused on biological responses to supplementation, including erythrocyte membrane fatty acid composition, prostaglandin sensitivity, and serum fatty acid analyses. Across the available evidence, the RCT did not demonstrate significant improvements in disability progression compared with oleic acid controls. Nevertheless, higher-dose linoleic acid supplementation was associated with shorter and less severe relapses in RRMS patients, but no apparent benefit was observed in patients with chronic progressive disease.

#### 3.2.2. Omega-3 Studies

Ten independent primary studies addressed omega-3 fatty acid supplementation in people with multiple sclerosis, corresponding to 12 publications because one study produced two additional reports addressing complementary biological outcomes (Table 2). Most studies were RCT, but there was also one non-randomized controlled study, one open-label pilot study, and one cohort study. Overall, the evidence predominantly involved patients with RRMS, whereas only one pilot trial specifically enrolled participants with major depressive disorder and another study included mixed MS phenotypes.

Intervention protocols showed considerable variability. Most studies investigated fish oil–derived omega-3 supplementation, whereas more recent studies evaluated purified omega-3 formulations either as stand-alone interventions or as adjuncts to DMTs, particularly fingolimod. The treatment duration ranged from one to 24 months, and there was substantial variation in omega-3 formulations, daily doses, comparator oils, and dietary background management. Comparator interventions included both biologically inert oils (e.g., mineral oil) and metabolically active vegetable oils, such as olive, corn, and soybean oil.

Further methodological heterogeneity was observed across the included studies. Recruitment ranged from small single-centre pilot studies to multicentre randomized trials, with differences in eligibility criteria, baseline disability, and disease duration. Adherence was commonly monitored through pill counts or patient diaries, but several studies also confirmed treatment adherence using serum or erythrocyte membrane fatty acid analyses. Background dietary control varied considerably, with some trials providing standardized dietary counselling or excluding participants with high habitual fish intake, whereas others imposed no dietary restrictions. Likewise, the integration of omega-3 supplementation with DMTs varied across studies, ranging from monotherapy designs to adjunctive interventions that required stable pharmacological treatment before enrolment. Additional heterogeneity also resulted from differences in the definitions of relapse, disability progression, and secondary outcome measures. Outcomes encompassed both clinical endpoints (e.g., disability, relapse activity, fatigue, depression, and quality of life) and biological measurements, such as inflammatory mediators, fatty acid composition, and mitochondrial function markers.

#### 3.2.3. Combined PUFA Studies

Four independent primary studies (corresponding to 8 publications) evaluating combined PUFA formulations were identified, comprising one phase II proof-of-concept study of PLP10 formulation [43], a subsequent phase III RCT of the same formulation (PLP10) [44], one independent RCT evaluating a modified PLP10 formulation (Neuroaspis PLP10) [45], and one RCT investigating hemp seed oil combined with evening primrose oil [46,47,48,49,50]. The study duration ranged from 6 to 30 months, with sample sizes ranging from 51 to 100 participants. All studies enrolled patients with RRMS (Table 3). Across all studies, baseline demographic and clinical characteristics were comparable between intervention and control groups, with participants generally presenting mild-to-moderate disability and established disease.

The combined PUFA interventions included formulations in which the PUFA component represented the principal therapeutic rationale, namely Neuroaspis PLP10 and hemp seed oil combined with evening primrose oil. The predefined eligibility criteria excluded broader multicomponent formulations in which the specific contribution of PUFAs could not be reasonably interpreted.

Despite differences in formulation, all interventions combined omega-3 and omega-6 fatty acids in predefined ratios, with two studies evaluating proprietary PLP10 formulations supplemented with antioxidant vitamins and one investigating naturally derived plant oils rich in PUFAs. Additionally, the studies shared several methodological characteristics, including RCT designs and the assessment of both clinical efficacy and biological response. Treatment adherence was monitored using complementary strategies, including returned medication containers, scheduled follow-up, dietary records, and, most importantly, objective assessment of erythrocyte membrane fatty acid composition, which confirmed biological incorporation of the supplemented PUFAs. The interventions were generally well tolerated, with no treatment-related serious adverse events reported. The principal challenge to long-term retention was the limited palatability of the liquid formulations, which accounted for most study withdrawals.

The outcome domains varied across studies, reflecting different research objectives. Clinical endpoints, including relapse activity and disability progression, were evaluated in all evidence sources, whereas magnetic resonance imaging (MRI) outcomes were only assessed in the PLP10 trials, demonstrating significant reductions in inflammatory lesion activity [43,44]. Functional performance was investigated in a dedicated Neuroaspis PLP10 study using detailed gait analysis and physical performance testing, which revealed improvements in gait quality and lower-limb muscular endurance, while walking speed, global muscle strength, and body composition remained unchanged [45].

Biological assessments extended beyond clinical outcomes and included erythrocyte membrane fatty acid composition, inflammatory cytokines, and lipid metabolism-related biomarkers. Combined PUFA supplementation consistently increased erythrocyte membrane PUFA content, supporting treatment adherence and biological activity. Hemp seed oil and evening primrose oil studies reported significant reductions in inflammatory cytokines/markers, particularly in interferon-gamma (IFN-γ) and interleukin 17 (IL-17), as well as increased IL-4 concentrations and decreased secretory phospholipase A2 (sPLA2) and delta-6 desaturase (FADS2) activity, and liver enzyme concentrations [46,47,48,49,50].

### 3.3. Characteristics of Included Systematic Reviews

Of the six included reviews (Table 4), five were conventional systematic reviews, one of which included a meta-analysis, and one was a Cochrane systematic review. All synthesized evidence from RCT, prospective studies, and observational studies to evaluate PUFA supplementation in people with MS. Considerable overlap in the primary studies was evident across the reviews, particularly among RCTs investigating omega-3 supplementation.

The systematic reviews covered the full spectrum of PUFA interventions represented in the included primary studies, with omega-3 fatty acids (particularly EPA and DHA) being the most frequently evaluated, followed by omega-6 fatty acids and combined PUFA formulations.

The reviews synthesized evidence from the primary studies’ main clinical and laboratory outcomes, which included relapse rate, disability progression, fatigue, quality of life, and inflammatory biomarkers. They covered all major MS phenotypes, although RRMS predominated across the evidence base, whereas SPMS and PPMS were under-represented and frequently included in mixed cohorts.

Most reviews performed methodological quality or risk-of-bias assessments of the included studies. However, formal evaluation of the certainty of evidence was uncommon, with only one review applying the GRADE framework. The available evidence was consistently considered limited by methodological weaknesses and heterogeneity among primary studies.

In general, the systematic reviews reached different conclusions across outcome domains. Whereas several reviews suggested that PUFA supplementation could improve relapse rate, fatigue, quality of life, and selected inflammatory biomarkers, evidence regarding disability progression remained inconsistent. Reviews that incorporated a more comprehensive methodological assessment, including the Cochrane review, concluded that the certainty of evidence was low and insufficient to support clinically meaningful benefits on disease progression.

### 3.4. Evidence Characterization

To provide additional context for interpreting the available literature, the primary-study publications (*n* = 24) were descriptively mapped according to predefined reporting and study characteristics. This exercise was intended to characterize the distribution of the evidence rather than to assess methodological quality or reporting quality. The mapping highlights the diversity of study designs, intervention reporting practices, and outcome domains investigated, as well as features that may influence the interpretability of the findings, such as the completeness of PUFA intervention reporting and the ability to attribute observed effects specifically to PUFA supplementation.

Table 5 summarizes the overall distribution of these characteristics across the 24 primary-study publications, while Supplementary Tables S5 and S6 provide the mapping framework and detailed publication-level mapping, respectively.

The descriptive mapping indicates that the included publications generally provided sufficient intervention detail to support reproducibility and interpretation, with most studies describing the PUFA formulation, dose, treatment duration, comparator condition, and multiple outcome domains. Partial PUFA-specific interpretability was mainly observed in studies evaluating more complex formulations or reporting secondary analyses, as detailed in Supplementary Table S6.

Table 6 summarizes the methodological characteristics of the systematic reviews included, while Supplementary Table S7 provides more detailed information. The reviews searched for literature published between 1962 and 2019 using multiple biomedical databases. Four reviews performed qualitative evidence synthesis, whereas two additionally conducted meta-analyses. Most reviews assessed the methodological quality or risk-of-bias of the included studies, most commonly using the Cochrane Risk of Bias tool, with only one review additionally applying the GRADE framework to assess the certainty of the evidence. Reported evidence assessments varied across reviews and are presented as originally reported, reflecting differences in appraisal methods and reporting approaches.

Funding sources and institutional support reported by the included independent evidence sources are summarized in Supplementary Table S8.

## 4. Discussion

### 4.1. Omega-6 Fatty Acids

The four primary studies investigating omega-6 supplementation are among the earliest clinical attempts to evaluate the therapeutic potential of PUFAs in MS. Together, these studies indicate that omega-6 supplementation induced reproducible biological changes but provided only modest and inconsistent clinical benefits. Improvements in erythrocyte membrane properties and, at higher doses, circulating PUFA concentrations were consistently observed, but these biological effects were not accompanied by convincing reductions in disability progression, while clinical benefits on relapse-related outcomes remained limited.

The available evidence also illustrates the diversity of research approaches used during this early phase of PUFA investigation. First, all studies were conducted between 1977 and 1983, before the development of current diagnostic criteria, standardized disability measures, MRI, and modern DMTs. Consequently, patient characterization, disease monitoring, and outcome assessment differ substantially from contemporary MS clinical trials. While the randomized trials primarily evaluated relapse activity and disability progression [28,29], longitudinal studies focused on biological responses to supplementation, particularly changes in erythrocyte membrane behavior [30,31]. These complementary approaches, despite their methodological differences, explored both clinical efficacy and underlying biological mechanisms.

Beyond these methodological differences, variations in intervention protocols further complicate the interpretation of clinical findings. Although all studies evaluated Naudicelle oil, containing LA with smaller amounts of γ-linolenic acid, the administered doses differed markedly, ranging from approximately 2.6 to 23 g/day of LA, and were delivered either as capsules or spread formulations. Notably, the only clinically beneficial findings were reported with the highest-dose intervention, whereas lower-dose regimens generally failed to produce measurable changes in serum fatty acid composition or clinical outcomes. This pattern suggests that dose may have influenced treatment response, but differences in formulation and study design preclude definitive conclusions regarding a dose–response relationship.

Another consistent observation was the apparent influence of disease phenotype. Patients with RRMS appeared to benefit more from supplementation than those with chronic progressive disease, in whom no meaningful clinical improvement was observed [28,29]. Although the original investigators attributed these differences to distinct pathological characteristics of the disease phenotypes, their explanations remained speculative.

Perhaps the most important finding emerging from these studies is the clear dissociation between biological activity and clinical efficacy. Omega-6 supplementation consistently modified laboratory biomarkers associated with membrane lipid composition, yet these changes translated into only limited clinical improvement [30,31]. This observation suggests that normalization of membrane-related biomarkers alone was insufficient to alter the clinical course of MS, highlighting the complexity of translating biological effects into meaningful therapeutic benefits.

The authors attributed the heterogeneous findings to insufficient dosing, delayed biological responses, and differences between relapsing and progressive disease, while also suggesting prostaglandin-mediated immunomodulation and membrane lipid abnormalities as potential mechanisms. These hypotheses reflected the scientific understanding of the period and stimulated further research, even though most were not subsequently confirmed.

Taken together, these pioneering studies demonstrated that isolated omega-6 supplementation was biologically active and generally well tolerated, but there was insufficient evidence of clinically meaningful disease modification. Despite their limitations, they established an important foundation for subsequent research investigating the role of PUFAs in MS.

The transition from isolated omega-6 supplementation to combined PUFA formulations reflects the advancement of both MS research and the understanding of fatty acid biology. Whereas the early studies focused primarily on correcting presumed abnormalities in LA metabolism and membrane function [30,31], later interventions targeted multiple complementary pathways involved in neuroinflammation, oxidative stress, and neuronal function through combinations of omega-3 and omega-6 fatty acids [43,44,45,48,49]. Important methodological advances, including contemporary diagnostic criteria, MRI, functional assessments, and immunological biomarkers, accompanied this conceptual shift. Moreover, combined formulations were increasingly evaluated as adjuncts to DMTs rather than stand-alone treatments [43,44,45]. These formulations also incorporated multiple fatty acids with complementary biological properties instead of relying predominantly on LA. Compared with the modest effects observed with isolated omega-6 supplementation, the combined PUFA studies reported improvements across a wider range of outcomes, including relapse activity, disability progression, MRI lesion burden, and functional performance [43,44,45,48]. However, these observations should be interpreted cautiously. The evidence remains limited to a small number of heterogeneous studies employing substantially different formulations and intervention strategies, making it difficult to determine whether the apparent improvement reflects the combined fatty acid composition, advances in study design, or differences in patient populations and concomitant therapies.

The conclusions of the included systematic reviews were generally consistent with the findings of the primary studies. In summary, they found insufficient evidence to support isolated omega-6 supplementation as an effective disease-modifying intervention although acknowledging possible benefits for selected relapse-related or symptomatic outcomes. The reviews consistently emphasized the methodological limitations of the early trials, including small sample sizes, heterogeneous interventions, and suboptimal dosing, requiring caution in interpreting the observed biological effects. Collectively, the primary studies and systematic reviews indicate that isolated omega-6 supplementation is biologically active, but there is limited evidence of clinically meaningful disease modification. Together with the evolving understanding of MS as a complex immune-mediated disease, these findings contributed to the progressive shift towards omega-3 and combined PUFA strategies.

### 4.2. Omega-3 Fatty Acids

The available evidence suggests that omega-3 fatty acid supplementation exerts consistent biological effects but limited and variable clinical benefits in MS patients. Across the included primary studies, supplementation was generally associated with favorable changes in fatty acid composition, including increased erythrocyte or serum EPA and DHA levels [32,34,37,39], as well as modulation of inflammatory pathways, reflected by reductions in several pro-inflammatory cytokines and eicosanoids [33,36,40,42]. Individual studies also reported improvements in oxidative stress markers and platelet mitochondrial membrane function [36,42]. These findings support the proposed anti-inflammatory and immunomodulatory effects of omega-3 fatty acids [12,14].

In contrast, improvements in clinically relevant outcomes were considerably less consistent. Most RCT did not demonstrate significant benefits on relapse activity, disability progression, or MRI outcomes, and more recent studies likewise failed to show significant improvements in patient-reported outcomes such as fatigue, physical activity, depression, or quality of life [27,39,40]. Some favorable findings were nevertheless reported in specific subgroups or exploratory analyses [27,32,42]. The current evidence suggests a gap between the measurable biological effects of omega-3 supplementation and their translation into consistent clinically meaningful benefits.

Several methodological factors may partly explain the inconsistent clinical findings. The included studies were highly heterogeneous in terms of participant characteristics, intervention protocols, treatment duration, background diet, concomitant DMTs, and outcome definitions, limiting direct comparisons between studies. Recruitment strategies ranged from small single-center pilot studies to multicenter RCT, while eligibility criteria varied considerably with respect to disease activity, disability status, and disease duration, potentially influencing baseline inflammatory burden and responsiveness to omega-3 supplementation [27,35,36,39,40,41].

Intervention protocols also differed substantially. Omega-3 fatty acids were evaluated both as stand-alone nutritional interventions and as adjuncts to DMTs, with treatment durations ranging from one month to two years. Background dietary management was inconsistently controlled, despite its potential influence on membrane lipid composition and inflammatory mediator synthesis. In addition, placebo formulations varied from metabolically inert mineral oils to metabolically active vegetable oils. In several studies included in this review, the control group received either olive oil or oleic acid, the predominant monounsaturated fatty acid in olive oil. In other studies, the control group received other plant oils, such as corn or soybean oil, which are also metabolically active. Therefore, the use of these oils may have reduced the biological contrast between intervention and control groups and may, at least partially, have contributed to the lack of statistically significant between-group differences observed in some clinical trials. Collectively, these methodological differences represent important sources of clinical heterogeneity that must be considered when interpreting the available evidence.

Several studies objectively confirmed adherence through serum or erythrocyte membrane fatty acid analyses, but successful biological incorporation of omega-3 fatty acids was not consistently accompanied by clinical improvement. Furthermore, definitions of relapse, disability progression, and sustained clinical worsening varied considerably across studies, limiting the comparability of efficacy endpoints and complicating evidence synthesis. Together, these observations suggest that inadequate biological exposure alone is unlikely to explain the inconsistent clinical findings.

Some methodological improvements should therefore be prioritized in future trials. Priorities include adequately powered RCT with longer follow-up, harmonized outcome definitions, appropriate intention-to-treat analyses, and the use of biologically inert placebo formulations [27,34,35,39], as well as better standardized omega-3 interventions with biologically justified EPA:DHA ratios, doses, and treatment strategies [27,40,42]. Addressing these considerations may minimize potential confounding and improve the interpretation of clinical efficacy.

Beyond methodological standardization, further research should clarify the biochemical mechanisms through which omega-3 fatty acids may influence disease activity and determine which mechanistic biomarkers are most closely associated with clinically meaningful outcomes. Despite consistent evidence of positive changes in inflammatory mediators, oxidative stress markers, and fatty acid composition, the relationship between these biological effects and improvements in disability, relapse activity, or patient-reported outcomes remains poorly understood [32,33,36,40,42]. Future studies should therefore integrate validated mechanistic biomarkers with clinical endpoints to determine the clinical relevance of these biological responses.

The evidence base should also be expanded to include diverse and clinically relevant patient populations. The available evidence is derived predominantly from adults with RRMS, providing limited information for progressive phenotypes, paediatric populations, patients with recent inflammatory activity, and individuals receiving contemporary high-efficacy DMTs [27,41,42]. Future research should also consider inter-individual biological variability, including inflammatory and metabolic profiles, while incorporating patient-centered outcomes such as fatigue, cognitive performance, and quality of life alongside conventional clinical endpoints [39]. Such approaches may help in identifying the patient subgroups most likely to benefit from omega-3 supplementation, thereby increasing the clinical applicability of future studies.

A comparison of the primary studies with the systematic reviews included in this scoping review indicates that the overall certainty of evidence for the clinical efficacy of omega-3 supplementation in MS remains largely unchanged. Recent primary studies are broadly consistent with previous evidence syntheses, showing no reproducible benefits on key clinical outcomes, including disability progression, relapse activity, and MRI lesion burden [54,55]. Occasional improvements in patient-reported outcomes, such as fatigue and quality of life, have been reported, but these findings remain inconsistent and do not greatly alter the overall interpretation of the evidence [51,53].

While the general clinical evidence has remained largely unchanged, recent studies have considerably expanded the understanding of the mechanisms underlying omega-3 supplementation. Beyond confirming favorable effects on fatty acid incorporation and selected inflammatory mediators, newer investigations have identified coordinated immunometabolic responses involving antioxidant, apoptotic, and T-cell regulatory pathways [42]. Emerging evidence also suggests potential neuroprotective effects reflected by neuro-ophthalmological measures, including retinal nerve fiber layer thickness and visual evoked potentials, extending previous observations based primarily on circulating inflammatory biomarkers [42]. Nevertheless, these mechanistic advances strengthen the biological plausibility of omega-3 supplementation but have not yet translated into consistent improvements in conventional clinical outcomes.

The persistent dissociation between biological activity and clinical efficacy is most likely due to both methodological and biological factors. The small sample sizes of many trials may have limited statistical power to detect modest but clinically relevant treatment effects, particularly for secondary endpoints and subgroup analyses, which typically require larger samples to provide reliable estimates. Relatively short intervention periods, heterogeneous populations and protocols, biologically active comparator oils, and challenges in maintaining effective blinding may further reduce the ability to detect treatment effects. In a chronic disease such as MS, relatively short follow-up may also be insufficient to determine whether an intervention has sustained effects on relapse activity or disability accumulation and therefore to establish long-term disease modification. Similarly, shorter trials have limited ability to detect uncommon or cumulative safety signals associated with prolonged supplementation. Consequently, the absence of major safety concerns or clinically meaningful effects over relatively short intervention periods should not be interpreted as definitive evidence regarding long-term safety or efficacy. However, the inconsistency of clinical findings may also reflect the limited magnitude of omega-3-mediated immunomodulation relative to the complex inflammatory and neurodegenerative processes underlying established MS. This may be particularly relevant in patients receiving contemporary DMTs, in whom additional benefits may be difficult to detect because of reduced residual inflammatory activity or potential ceiling effects. Thus, the absence of reproducible clinical benefit cannot be attributed solely to trial design; it should also be interpreted in the context of MS pathophysiology and the inherent challenges of evaluating nutritional interventions. Addressing these uncertainties will require adequately powered, longer-duration trials using standardized formulations, inert comparators, harmonized clinical endpoints, and integrated mechanistic biomarkers across more diverse MS populations. Evidence regarding differential responses across MS subpopulations was limited. Most recent trials focused predominantly on RRMS, while studies including other or mixed phenotypes did not provide sufficiently powered comparative subgroup analyses to determine whether PUFA effects differ according to MS phenotype or other patient characteristics.

The global evidence suggests that omega-3 supplementation exerts reproducible biological effects; however, the conditions under which these responses translate into clinically meaningful benefits remain uncertain. Accordingly, the scientific focus has progressively shifted from establishing biological activity to identifying the formulations, patient characteristics, treatment contexts, and outcome measures that are most likely to reveal clinically relevant effects. This unresolved biological-clinical gap also provides a rationale for the subsequent investigation of combined PUFA formulations designed to target complementary lipid-mediated pathways.

The concomitant use of PUFA supplementation with modern DMTs remains poorly explored. Adding omega-3 to fingolimod did not improve relapse rate, disability, or cytokine profiles [41], whereas more recent findings from a multicomponent regimen including omega-3 and fingolimod suggested favorable immunological and neuro-ophthalmological effects [42]. However, the specific contribution of omega-3 could not be isolated in the latter study, and current evidence remains insufficient to establish whether PUFA supplementation modifies the therapeutic effects of DMTs.

### 4.3. Combined Fatty Acids

Combined PUFA interventions differed fundamentally from previous studies in that they were conceived as rationally designed formulations that targeted multiple components of MS pathophysiology simultaneously. Rather than viewing individual PUFAs primarily as isolated nutritional supplements or structural membrane constituents, the authors increasingly proposed that specific combinations of omega-3 and omega-6 fatty acids, together with antioxidant vitamins, could act synergistically on interconnected pathways involved in neuroinflammation, oxidative stress, and neurodegeneration. This conceptual evolution shifted the research focus from simple nutritional supplementation to a systems-based therapeutic approach in which individual components were selected to provide complementary biological functions.

The rationale extended beyond increasing total PUFA intake by combining complementary biological functions. Omega-3 fatty acids were intended to promote inflammation resolution and specialized pro-resolving mediator synthesis [43,49], whereas gamma-linolenic acid (GLA) was incorporated to support alternative anti-inflammatory lipid pathways [44,47,48]. Several formulations also included downstream metabolic intermediates and antioxidant vitamins to compensate for proposed alterations in endogenous PUFA metabolism while protecting membrane lipids from peroxidation during prolonged supplementation [46,47,48,49,50].

Consistent with the methodological evolution observed in the later omega-3 studies, an important finding emerging from these studies is the progressive expansion of outcome assessment. While early studies focused primarily on relapse frequency and disability scales, subsequent investigations incorporated MRI measures, quantitative gait analysis, erythrocyte membrane lipidomics, and immunometabolic biomarkers. This multidimensional assessment reflects an attempt to establish mechanistic links between fatty acid incorporation, biological responses, and clinical outcomes, providing greater insight into the potential modes of action of combined PUFA formulations.

Despite these methodological advances, the evidence base remains limited by the small number of available studies, modest sample sizes, heterogeneity of formulations and outcomes, and the inclusion of only patients with RRMS. Attrition related to the palatability of liquid formulations reduced statistical power in some early trials [44,45,48].

The systematic reviews were concordant with the primary studies regarding favorable biological activity, safety profiles, and relapse-related findings of combined PUFA formulations. However, they interpreted these findings more cautiously because the available evidence was derived from a limited number of heterogeneous trials and therefore remained insufficient to support firm clinical recommendations. The included systematic reviews consistently highlighted the limited quantity of evidence, methodological heterogeneity, and relatively small sample sizes as the principal factors limiting confidence in the observed effects [51,52,53,54].

Importantly, the more cautious conclusions of the systematic reviews are largely due to differences in the level of synthesized evidence rather than direct disagreement with the primary studies. While the individual trials consistently reported favorable clinical and biological outcomes for combined PUFA formulations, the reviews prioritized the overall certainty and reproducibility of the evidence, concluding that larger, well-designed RCT are required before these interventions can be incorporated into routine clinical practice. Thus, the current uncertainty is primarily about the strength of the evidence rather than the inconsistency in the observed direction of effect.

### 4.4. Systematic Reviews

The six systematic reviews included in this scoping review provide a complementary perspective on the evolution of PUFA research in MS. Published between 2017 and 2022, they progressed from evaluating isolated omega-3 and omega-6 interventions to incorporating combined PUFA formulations and broader mechanistic outcomes, mirroring the progression of primary research. Although individual primary studies frequently reported biologically plausible and, in some cases, clinically promising findings, the reviews consistently interpreted these results within the broader context of methodological heterogeneity, limited sample sizes, and variability in PUFA formulations and outcome measures. Rather than contradicting the primary evidence, they highlighted the limited certainty of clinical benefit and the need for adequately powered and methodologically harmonized trials. The convergence of conclusions across successive reviews suggests that the principal challenges are the limited number of studies as well as the ongoing heterogeneity of interventions and outcome measures, which limit evidence synthesis and definitive clinical recommendations.

### 4.5. Evidence Characterization

The descriptive evidence mapping indicates that research on PUFA supplementation in MS has developed into a predominantly intervention-based field, characterized by randomized designs, defined comparator conditions, and generally well-described supplementation protocols. Most publications reported dose and treatment duration, supporting reproducibility at the intervention level. The frequent assessment of laboratory, lipid-related, imaging, functional, clinical, and safety outcomes further reflect a progressive broadening of the research framework beyond conventional relapses and disability endpoints. This pattern is consistent with the evolution observed across the omega-6, omega-3, and combined PUFA literature, with later studies increasingly attempting to link fatty acid incorporation and mechanistic responses to clinically relevant outcomes.

Importantly, these descriptive characteristics should not be interpreted as evidence of uniformly high methodological quality or clinical efficacy. Intervention clarity indicates whether the administered PUFA formulation was sufficiently characterized, whereas PUFA-specific interpretability reflects whether the reported findings are reasonably related to a defined PUFA intervention. Neither classification evaluates the strength of causal inference, risk of bias, or certainty of evidence. Most publications were interpretable at the PUFA level, while partial interpretability was mainly associated with multicomponent interventions, dietary co-interventions, or combined pharmacological regimens, which limited attribution of observed effects to PUFA supplementation alone.

The predominance of multidomain outcome assessment also indicates increasing methodological range, but not necessarily greater comparability across studies. As demonstrated in the intervention-specific sections, differences in PUFA composition, dose, formulation, treatment duration, concomitant therapy, participant characteristics, and outcome definitions continue to restrict cross-study synthesis. Thus, clearer intervention reporting has improved the reproducibility and interpretability of individual protocols without resolving the clinical and methodological heterogeneity that underpins the inconsistent conclusions regarding efficacy. This heterogeneity also precludes conclusions regarding optimal supplementation strategies. Accordingly, precision supplementation, specific PUFA ratios, and combined formulations should be regarded as priorities for future investigation rather than as established or emerging therapeutic approaches.

The methodological characterization of the included systematic reviews supports the same distinction between study design and evidentiary certainty. Most reviews applied methodological quality or risk-of-bias assessments, predominantly using Cochrane-based tools, with only one also evaluating certainty of evidence using the GRADE framework, and appraisal methods varied across reviews. Their assessments were therefore not directly comparable. Nevertheless, the reviews consistently interpreted the evidence cautiously, indicating that the predominance of randomized designs and generally adequate intervention reporting were insufficient to overcome limitations in sample size, methodological consistency, and reproducibility across studies. These findings reinforce that randomization, intervention clarity, and multidomain assessment are important characteristics of the evidence base, but they alone are insufficient to establish high-certainty evidence or clinically meaningful efficacy.

Funding across the included independent evidence sources was heterogeneous (Supplementary Table S8). Most studies were supported by governmental agencies, universities, or multiple sclerosis charities, whereas manufacturer involvement was generally limited to supplying intervention products or placebo formulations. Mixed public and industry support or manufacturer involvement was reported in a limited number of studies [28,29,32,34,35,39,45], while several more recent studies explicitly declared no external funding. Funding sources and industry involvement may contribute to the assessment of risk of bias or certainty of evidence, which was out of scope for this scoping review. Accordingly, these data are presented solely as descriptive characteristics of the evidence base.

**Figure 2:**
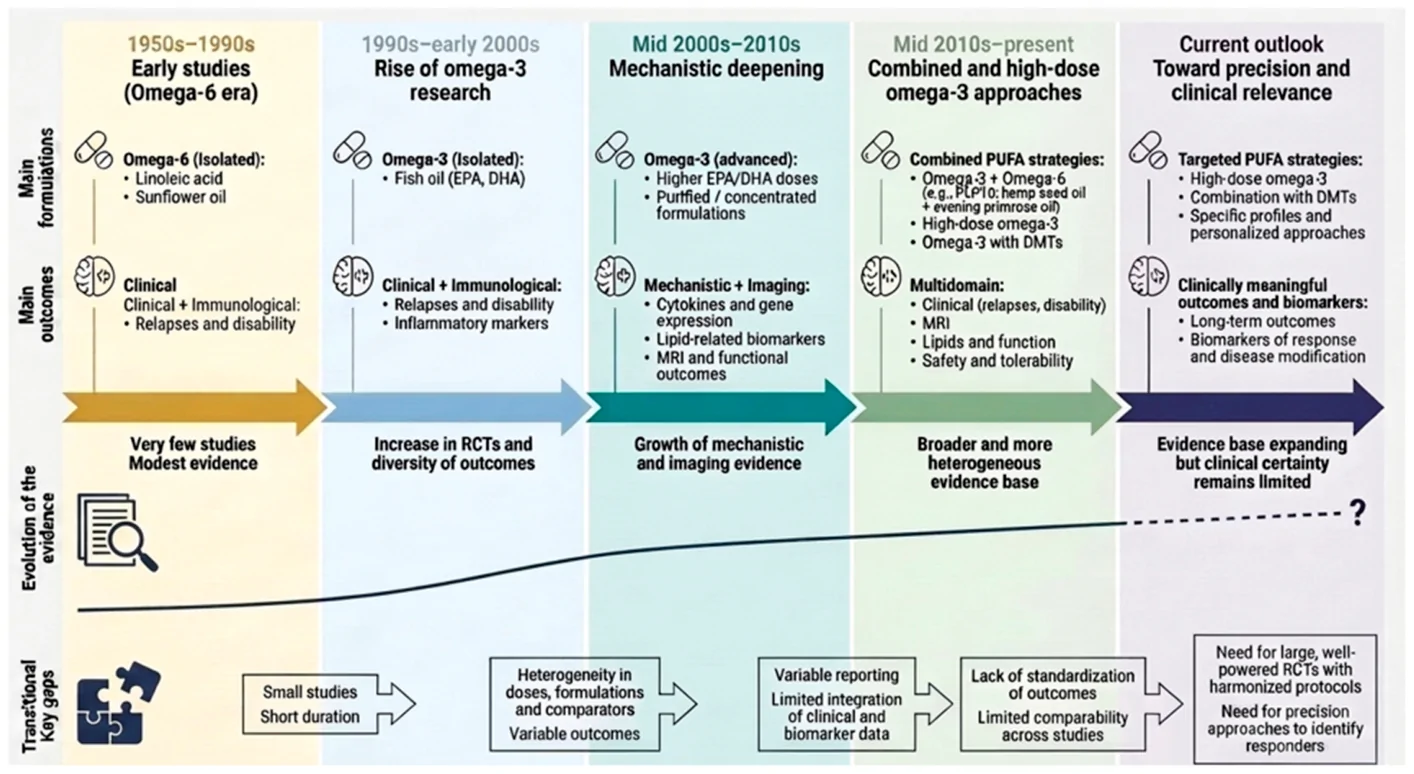
Chronological evolution of research on Polyunsaturated Fatty Acid (PUFA) supplementation in Multiple Sclerosis (created by the authors using NotebookLM image generation and subsequently refined for scientific accuracy). DHA: Docosahexaenoic Acid; DMTs: Disease-Modifying Therapies; EPA: Eicosapentaenoic Acid; MRI: Magnetic Resonance Imaging; RCTs: Randomized Clinical Trials.

Figure 2 summarizes the overall evolution of PUFA research in MS, including the chronological progression of intervention strategies, the expansion of research objectives and outcome domains, the development of the evidence base, and the major knowledge gaps that remain to be addressed.

Beyond the six systematic reviews published between 2017 and 2022, the present scoping review extends the evidence base by incorporating more recent studies, mapping publication families and independent evidence sources, comparing the evolution of PUFA formulations, and identifying persistent evidence gaps, particularly regarding the clinical relevance of biological effects and PUFA supplementation alongside contemporary DMTs.

### 4.6. Limitations

This scoping review has some limitations. Despite the comprehensive search strategy, relevant studies may have been overlooked due to variations in indexing, terminology, and reporting of PUFA interventions across databases. In addition, five potentially eligible reports could not be retrieved and thus could not be assessed for eligibility, which may have affected the completeness of the evidence mapping. Moreover, Embase was not searched due to unavailable access. Although Embase is an important database for clinical and pharmaceutical research, our search strategy covered five complementary databases, three clinical trial registries, and reference-list screening, aiming for a thorough and comprehensive evidence retrieval. Nevertheless, the absence of Embase may have resulted in some relevant records being missed.

The evidence mapping was intended to provide a structured overview of the characteristics of the available literature rather than a formal assessment of methodological quality or certainty of evidence. Consequently, the mapping classifications should be interpreted descriptively. A domain classified as unclear or incompletely reported may reflect reporting limitations rather than deficiencies in study conduct, whereas apparently complete reporting of intervention characteristics or outcomes does not necessarily indicate methodological rigor.

As this was a scoping review, no formal critical appraisal or risk-of-bias assessment of the included primary studies was undertaken. Consequently, the mapped evidence should be interpreted with appropriate caution, as the methodological quality and certainty of the mapped evidence could not be established within this study. Despite the broad scope of this review, which enabled a comprehensive overview of the literature on PUFA supplementation in MS, it necessarily limited the depth of analysis devoted to individual interventions and specific clinical questions. Accordingly, the findings provide an overview of the current evidence landscape but should not be used to support clinical recommendations or inform treatment guidelines regarding PUFA supplementation in MS.

## 5. Conclusions

This scoping review provides a comprehensive overview of the evidence on PUFA supplementation in MS, mapping the evolution of research from early studies of isolated omega-6 fatty acids to more recent investigations into omega-3 and combined PUFA formulations. While substantial biological and mechanistic advances have been achieved, the available evidence remains heterogeneous and insufficient to support definitive conclusions regarding clinical efficacy or routine therapeutic use.

Beyond summarizing the available literature, this review provides a structured characterization of the evidence base through descriptive evidence mapping, highlighting methodological trends, reporting practices, knowledge gaps, and areas that require further investigation. By integrating evidence from both primary studies and systematic reviews, it offers a framework for designing future well-powered, methodologically harmonized clinical trials and more focused evidence syntheses addressing specific PUFA formulations and clinically relevant outcomes for MS.

## Figures and Tables

**Table 1:** Characteristics of the included omega-6 primary studies.

Evidence Source	Design	Population (*n*)	Intervention/Exposure	Comparator	Duration	Clinical Outcomes	Biological Outcomes
Bates, 1977 [29]	Double-blind RCT	Chronic progressive MS (152)	Group A: Naudicelle oil (3.42 g linoleic acid + 360 mg linolenic acid daily); Group C: 11.5 g linoleic acid daily in a spread	Group B: Oleic acid capsules; Group D: 4 g oleic acid daily in a spread	2 years	No significant difference between groups in *n*. º of patients improved, deteriorated, or unchanged; no difference in attack rates or relapse scores	NR
Bates, 1978 [28]	Double-blind RCT	Acute remitting MS (RRMS)(116)	Group A: Naudicelle capsules (2.92 g linoleic acid + 0.34 g γ-linolenic acid daily); Group C: 23 g linoleic acid daily in a spread	Group B: 4 g oleic acid daily (capsules); Group D: 16 g oleic acid daily (spread)	2 years	No significant difference in clinical deterioration or attack frequency; exacerbations were significantly shorter and less severe in Group C compared to controls; no effect on overall disability	Significant increase in serum linoleic and arachidonic acids for Group C; no significant increase in PUFA percentages for Group A
Field, 1978 [31]	Longitudinal observational study	Established MS (53)	γ-linolenate (Naudicelle capsules: 2.664 g linoleic acid and 413.4 mg γ-linolenic acid daily)	Pre-treatment baseline	Up to 3 years	NR; some objective improvement noted	Reversion of RBC electrophoretic mobility (normalization) in response to linoleic and arachidonic acids; significant increase in sensitivity to PGE2 on RBC mobility
Field, 1983 [30]	Longitudinal observational study	Ambulant MS (28)	γ-linolenate (Naudicelle capsules: 300 mg γ-linolenic acid daily) and low animal fat diet	Pre-treatment baseline and normal control reactivity	6–24 months (up to 2 years or more)	NR; noted that clinical trials should ideally begin after 2 years when membrane reactivity of RBC is restored	RBC membrane reactivity (E-UFA test) reversed from positive to negative (normal); PGE2 normalized after 21–24 months of treatment

ACTH: Adrenocorticotropic Hormone; E-UFA: Erythrocyte-Unsaturated Fatty Acid (test); MS: Multiple Sclerosis; NR: Not Reported; PGE2: Prostaglandin E2; PUFA: Polyunsaturated Fatty Acid; RBC: Red Blood Cell; RCT: Randomized Controlled Trial.

**Table 2:** Characteristics of the included omega-3 primary studies.

Evidence Source ^1^	Study Design	Population	Intervention	Comparator	Duration	Clinical Outcomes	Biological Outcomes
*Omega-3 monotherapy*							
Bates et al., 1989 [32]	Double-blind controlled trial	RRMS (*n* = 312)	20 capsules/day *n*-3 PUFAs (MaxEPA: 1.71 g EPA, 1.14 g DHA) + *n*-6 dietary advice	Olive oil (oleic acid) + *n*-6 dietary advice	2 years	No significant differences in relapse frequency, duration or severity	Increased serum and adipose tissue EPA and DHA concentrations
Gallai et al., 1995 [33]	Non-randomized controlled trial	RRMS (*n* = 20); healthy controls (*n* = 15)	6 g/day fish body oil (ESAPENT: 51% EPA, 31% DHA)	Age-matched healthy controls	6 months	Trend towards reduced acute relapse activity	Significant decreases in IL-1β, TNF-α, IL-2, IFN-γ, PGE_2_ and LTB4 in stimulated PBMCs; reduced serum soluble IL-2 receptors
Shinto et al., 2009 [34]	Open-label pilot study	RRMS (*n* = 10)	9.6 g/day fish oil (2.9 g EPA, 1.9 g DHA)	Baseline	3 months	No significant change in quality of life; well tolerated	Increased RBC membrane EPA and DHA; reduced MMP-9 secretion
Torkildsen et al., 2012 [35]	Double-blind RCT (OFAMS)	RRMS (*n* = 92)	Omega-3 FAs (1350 mg EPA, 850 mg DHA daily)	Corn oil	24 months	No significant improvement in MRI activity, relapse rate, EDSS or fatigue	Increased serum omega-3 concentrations
Ramirez-Ramirez et al., 2013 [36] Additional reports ^2^: Sorto-Gomez et al., 2016 [37]; Torres-Sánchez et al., 2018 [38]	Double-blind RCT	RRMS (*n* = 50)	4 g/day fish oil (0.8 g EPA, 1.6 g DHA)	Olive oil	12 months	No significant differences in EDSS or annualized relapse rate	Significant reductions in IL-1β, TNF-α, IL-6 and nitric oxide metabolites; increased serum omega-3, reduced AA/EPA ratio; no difference in glutathione reductase activity or glutathione levels; improved platelet mitochondrial membrane fluidity and decreased hydrolytic ATP synthase activity
Shinto et al., 2016 [39]	Double-blind RCT (pilot)	Mixed MS (RRMS and progressive) with MDD (*n* = 39)	6 g/day fish oil (1.95 g EPA, 1.35 g DHA)	Placebo (soybean oil with 1% fish oil)	3 months	No significant group difference in depression improvement (MADRS, BDI) or quality of life	Significant increase in RBC membrane EPA and DHA
Golabi et al., 2025 [27]	Double-blind RCT	MS (*n* = 68)	2000 mg/day omega-3 FA (360 mg EPA, 240 mg DHA)	Mineral oil	12 weeks	No significant effect on chronic fatigue or physical activity	No significant changes in hs-CRP or BDNF
Majdinasab et al., 2026 [40]	Double-blind RCT	RRMS (*n* = 60)	1 g daily Omega-3	1 g daily Royal Jelly	1 month	NR	Significant decrease in TGF-β and IFN-γ and increase in IL-4
*Omega-3 as adjunctive therapy*							
Zandi-Esfahan et al., 2017 [41]	Double-blind RCT	RRMS (*n* = 50)	1 g/day fish oil add-on to Fingolimod	Paraffin oil + fingolimod	12 months	No significant differences in EDSS or relapse rate	No significant differences in IL-17, IFN-γ, IL-4 or TGF-β
Ahmed Merza Mohammed et al., 2026 [42]	Cohort study nested cross-sectional	RRMS (*n* = 80)	Omega-3 added to Fingolimod + Metformin + Fluoxetine	Fingolimod monotherapy or combo without Omega-3	NR	Improved neuro-ophthalmological outcomes (RNFL thickness and VEP parameters)	Reduced pro-inflammatory cytokines (IL-6, TNF-α); increased IL-10; higher BCL2, NRF2, SOD2, and AMPK2 expression; reduced Th1/Th17 ratio

AA: Arachidonic Acid; ABCA1: ATP-binding cassette transporter A1; AMPK2: AMP-activated protein kinase 2; ApoA1: Apolipoprotein A1; BCL2: anti-apoptotic gene; BDI: Beck Depression Inventory; BDNF: Brain-Derived Neurotrophic Factor; CHROME: Cholesterol Homeostasis Regulator Of miRNA Expression; DHA: Docosahexaenoic Acid; EDSS: Expanded Disability Status Scale; EPA: Eicosapentaenoic Acid; hs-CRP: high-sensitivity *C*-Reactive Protein; IFN-γ: Interferon-gamma; IL: Interleukin; LTB4: Leukotriene B4; MADRS: Montgomery–Asberg Depression Rating Scale; MDD: Major Depressive Disorder; MMP-9: Matrix Metalloproteinase-9; MRI: Magnetic Resonance Imaging; NR: not reported; NRF2: antioxidant-related gene; OFAMS: Omega-3 Fatty Acids in Multiple Sclerosis Study; PBMCs: Peripheral Blood Mononuclear Cells; PGE2: Prostaglandin E2; PUFA: Polyunsaturated Fatty Acid; RBC: Red Blood Cell; RCT: Randomized Controlled Trial; RNFL: Retinal Nerve Fiber Layer; RRMS: Relapsing–Remitting Multiple Sclerosis; S1PR: Sphingosine 1-Phosphate Receptor; SOD2: Superoxide Dismutase 2; TGF-β: Transforming Growth Factor-beta; TGs: Triglycerides; Th1/Th17: Type 1 and Type 17 helper T cell subsets; TNF-α: Tumor Necrosis Factor-alpha; VEP: Visual Evoked Potentials. ^1^ Evidence sources are represented by the primary publication. Where multiple publications originated from the same study population, these are indicated as additional reports and were considered a single evidence source during data synthesis. ^2^ Additional reports contributed supplementary evidence on erythrocyte fatty acid composition (Sorto-Gomez et al., 2016) [37] and platelet mitochondrial membrane fluidity and ATP synthase activity (Torres-Sánchez et al., 2018) [38].

**Table 3:** Characteristics of the included combined PUFA primary studies.

Evidence Source	Study Design	Population	Intervention	Comparator	Duration	Clinical Outcomes	Biological Outcomes
*PLP10 formulation*							
Pantzaris et al., 2013 [44]	Phase II proof-of-concept RCT (2013)	RRMS (*n* = 80)	PUFA mix (omega-3 [EPA 1650 mg, DHA 4650 mg] and omega-6 [GLA 2000 mg, LA 3850 mg] at 1:1 wt/wt) + Vitamins A, E (Group A) PLP10 (omega-3 [EPA 1650 mg, DHA 4650 mg] and omega-6 [GLA 2000 mg, LA 3850 mg] at 1:1 wt/wt) + Vitamins A, E, and γ-tocopherol 760 mg (Group B) γ-tocopherol 760 mg (Group C)	Virgin olive oil	30 months	Reduced ARR; delayed disability progression; safe and well tolerated	Reduced risk of new/enlarging T2 lesions; compliance confirmed by RBC membrane fatty acid composition
Pantzaris et al., 2022 [43]	Multicenter Phase III RCT (2022)	RRMS (*n* = 61 receiving IFN-β)	PLP10 (omega-3 [EPA 1650 mg, DHA 4650 mg] and omega-6 [GLA 2000 mg, LA 3850 mg] at 1:1 wt/wt) + Vitamins A, E, and γ-tocopherol 760 mg	Virgin olive oil	30 months	Reduced ARR; delayed disability progression; safe and well tolerated	Reduced risk of new/enlarging T2 lesions and Gd-enhancing lesions
*Neuroaspis PLP10*							
Aristotelous et al., 2021 [45]	Double-blind RCT	RRMS (*n* = 51)	Neuroaspis PLP10 (mixture of omega-3 and omega-6 PUFAs: EPA 810 mg, DHA 4140 mg, GLA 1800 mg, LA 3150 mg) + Vitamins A, E, and γ-tocopherol	Virgin olive oil	24 months	Improved gait (GDI), balance and muscular endurance (STS-60); no significant effect on 6MWT or TUG	No significant changes in body composition or anthropometric measures
*Hemp seed oil + evening primrose oil*							
Rezapour-Firouzi et al., 2013 [49] *Additional reports*: Rezapour-Firouzi et al., 2014 [50] Rezapour-Firouzi et al., 2013 [46] Rezapour-Firouzi et al., 2014 [47] Rezapour-Firouzi et al., 2015 [48]	Double-blind RCT	RRMS (*n* = 100)	Hemp seed oil + evening primrose oil (9:1, 18–21 g/day) with (Group A) or without (Group C) Hot-nature diet	Olive oil	6 months	Improved EDSS and relapse rate in intervention groups; deterioration in control group	Increased RBC membrane PUFAs (EPA, AA); increased IL-4 and reduced IL-17 and IFN-γ; reduced ALT and AST; modulation of FADS2, PLA2 and Δ6-desaturase activity

6MWT: Six-Minute Walk Test; AA: Arachidonic Acid; ALT: Alanine aminotransferase; ARR: Annualized Relapse Rate; AST: Aspartate aminotransferase; D6D: Delta-6 Desaturase; DHA: Docosahexaenoic Acid; EDSS: Expanded Disability Status Scale; EPA: Eicosapentaenoic Acid; FADS2: Fatty Acid Desaturase 2 gene; GDI: Gait Deviation Index; GLA: Gamma-Linolenic Acid; IFN: Interferon; IL: Interleukin; LA: Linoleic Acid; RBC: Red Blood Cell; RCT: Randomized Controlled Trial; RRMS: Relapsing–Remitting Multiple Sclerosis; sPLA2: Secretory Phospholipase A2; STS: Sit-to-Stand test; TUG: Timed Up and Go test.

**Table 4:** Characteristics of the included systematic reviews.

Evidence Source	Review Type	Studies Included	PUFA Interventions Covered	Main Outcomes
AlAmmar, 2021 [51]	Systematic review	7 interventional or prospective observational studies	Omega-3 fatty acids (EPA, DPA, DHA) and fish oil supplementation	Beneficial effects on improving quality of life, reducing relapsing rate, and improving inflammatory markers and glutathione reductase
Bagur, 2017 [52]	Systematic review	47 articles (27 clinical trials and 20 observational studies)	PUFAs (omega-3), EPA, DHA, linoleic acid, gamma-linolenic acid, and various oils (cod liver, sesame, hemp, evening primrose, fish)	Decreased relapse rate, fatigue, and risk of developing the disease; improved EDSS; decreased inflammatory markers (IL-6, CRP) and oxidative stress
Harirchian, 2022 [53]	Systematic review	15 clinical trials	Fish oil (EPA, DHA) and vegetable oils (evening primrose, hemp seed, olive)	Small effect on disability for fish oil; medium to large effect on relapse rate for evening primrose and hemp seed oils; improvements in fatigue and quality of life
Hempel, 2017 [54]	Systematic review	37 RCTs (including 9 studies on fatty acid supplementation)	PUFA intake (omega-3, omega-6), EPA, DHA, GLA, linoleic acid, and various oils (sunflower, borage, fish)	No statistically significant effect identified for fatty acid supplementation on MS progression or mean EDSS scores
Parks, 2020 [55]	Cochrane Review (Systematic review)	30 trials (including 11 trials on fatty acid supplementation)	PUFAs (omega-3, omega-6), MUFAs, and specific oil formulations	Assessed relapses (rate, duration, severity), disability progression (EDSS, MSFC), global impression of deterioration or enhancing lesions (MRI) provide little to no difference in MS. Low rates of serious adverse events were reported
Sedighiyan, 2019 [56]	Systematic review and meta-analysis	4 trials	Omega-3 supplementation (EPA, DHA), fish oil	No significant effect on EDSS, IL-1β, or IL-6; significant reduction in TNF-α concentration compared to placebo

CRP: *C*-reactive protein; DHA: Docosahexaenoic Acid; DPA: Docosapentaenoic Acid; EDSS: Expanded Disability Status Scale; EPA: Eicosapentaenoic Acid; GLA: Gamma-Linolenic Acid; IL-1β: Interleukin-1 beta; IL-6: Interleukin-6; MRI: Magnetic Resonance Imaging; MS: Multiple Sclerosis; MSFC: Multiple Sclerosis Functional Composite; PUFA: Polyunsaturated Fatty Acid; RCTs: Randomized Controlled Trials; TNF-α: Tumor Necrosis Factor-Alpha.

**Table 5:** Distribution of the evidence mapping characteristics across the 24 primary-study publications.

Mapping Item	Category	*n* (%)
Study design	Randomized controlled trial	19 (79.2)
	Longitudinal observational study	2 (8.3)
	Non-randomized controlled trial	1 (4.2)
	Open-label pilot study	1 (4.2)
	Cohort study (nested cross-sectional)	1 (4.2)
PUFA supplement description	Clear	22 (91.7)
	Partially clear	2 (8.3)
Dose and duration reporting	Both reported	23 (95.8)
	Neither reported	1 (4.2)
Comparator	Independent comparator	20 (83.3)
	Within-subject comparison (baseline)	4 (16.7)
Outcome domain(s)	Clinical	1 (4.2)
	Laboratory-immunological	4 (16.7)
	Multiple domains	19 (79.2)
PUFA-specific interpretability	Yes	18 (75.0)
	Partial	6 (25.0)

PUFA: Polyunsaturated fatty acid. Note: Frequencies refer to primary-study publications (*n* = 24) rather than independent evidence sources.

**Table 6:** Methodological appraisal characteristics of systematic reviews.

Mapping Item	Category	*n* (%)
Risk of bias assessment	Cochrane RoB	3 (50.0)
	Cochrane RoB + GRADE	1 (16.7)
	Not reported	2 (33.3)
Reported evidence assessment	Reported	4 (66.7)
	Not reported	2 (33.3)

RoB: Risk of Bias; GRADE: Grading of Recommendations Assessment, Development, and Evaluation.

## Data Availability

No new data were created or analyzed in this study. Data sharing is not applicable.

## Supplementary Materials

The following supporting information can be downloaded at: https://www.mdpi.com/article/10.3390/nu18172915/s1, Table S1: Operational eligibility criteria used for study selection; Table S2: Electronic search strategies for each database and trial registry; Table S3: Structured data charting form and core variables; Table S4: Full-text articles excluded after eligibility assessment and reasons for exclusion; Table S5: Evidence mapping interpretability; Table S6: Evidence mapping of study reporting; Table S7: Methodological characteristics of the systematic reviews; Table S8: Funding sources and institutional support reported by the included independent evidence sources; File S1: Operational framework for AI-assisted screening and data charting.

## Abbreviations

The following abbreviations are used in this manuscript:

6MWT	Six-Minute Walk Test
AA	Arachidonic Acid
ABCA1	ATP-binding cassette transporter A1
ACTH	Adrenocorticotropic Hormone
ALA	α-Linolenic Acid
ALT	Alanine aminotransferase
AMPK2	AMP-activated protein kinase 2
ApoA1	Apolipoprotein A1
ARR	Annualized Relapse Rate
AST	Aspartate aminotransferase
BCL2	Anti-apoptotic gene
BDI	Beck Depression Inventory
BDNF	Brain-Derived Neurotrophic Factor
CIS	Clinically Isolated Syndrome
CHROME	Cholesterol Homeostasis Regulator of miRNA Expression
CRP	*C*-reactive protein
D6D	Delta-6 Desaturase
DHA	Docosahexaenoic Acid
DMTs	Disease-Modifying Therapies
DPA	Docosapentaenoic Acid
EDSS	Expanded Disability Status Scale
EPA	Eicosapentaenoic Acid
EU CTR/EudraCT	European Union Clinical Trials Register
E-UFA	Erythrocyte-Unsaturated Fatty Acid
FADS2	Fatty Acid Desaturase 2 gene
GDI	Gait Deviation Index
GLA	Gamma-Linolenic Acid
GRADE	GRADE: Grading of Recommendations Assessment, Development, and Evaluation
hs-CRP	high-sensitivity *C*-Reactive Protein
IFN-γ	Interferon-gamma
IL	Interleukin
IL-1β	Interleukin-1 beta
JBI	Joanna Briggs Institute
LA	Linoleic Acid
LLM	Large Language Model
LTB4	Leukotriene B4
MADRS	Montgomery–Asberg Depression Rating Scale
MDD	Major Depressive Disorder
MMP-9	Matrix Metalloproteinase-9
MRI	Magnetic Resonance Imaging
MS	Multiple Sclerosis
MSFC	Multiple Sclerosis Functional Composite
NR	Not Reported
NRF2	antioxidant-related gene
OFAMS	Omega-3 Fatty Acids in Multiple Sclerosis Study
OSF	Open Science Framework
PBMCs	Peripheral Blood Mononuclear Cells
PCC	Participants–Concept–Context
PGE2	Prostaglandin E2
PPMS	Primary Progressive Multiple Sclerosis
PRISMA-ScR	Preferred Reporting Items for Systematic Reviews and Meta-Analyses extension for Scoping Reviews
PUFA	Polyunsaturated Fatty Acid
RBC	Red Blood Cell
RCT	Randomized Clinical Trial
RNFL	Retinal Nerve Fiber Layer
RoB	Risk of Bias
RRMS	Relapsing–Remitting Multiple Sclerosis
S1P	Sphingosine-1-Phosphate
S1PR	Sphingosine 1-Phosphate Receptor
SOD2	Superoxide Dismutase 2
sPLA2	Secretory Phospholipase A2
SPMS	Secondary Progressive Multiple Sclerosis
STS	Sit-to-Stand test
TGF-β	Transforming Growth Factor-beta
TGs	Triglycerides
Th1/Th17	Type 1 and Type 17 helper T cell subsets
TNF-α	Tumour Necrosis Factor-alpha
TUG	Timed Up and Go test
VEP	Visual Evoked Potentials
WHO IC-TRP	World Health Organization International Clinical Trials Registry Platform
