# Supplementary material for: Polyunsaturated Fatty Acid Supplementation in Multiple Sclerosis Management: A Scoping Review
Source: Nutrients. 2026 Sep 5;18(17):2915. doi: 10.3390/nu18172915 (PMC13567611; doi:10.3390/nu18172915)
Supplement: Supplementary file 1 [file nutrients-18-02915-s001.zip › Supplement Tables.pdf]

Supplementary Tables

Table S1. Operational eligibility criteria used for study selection

Criterion	Inclusion criteria	Exclusion criteria
**Participants**	Humans diagnosed with MS (all phenotypes), CIS, or a first demyelinating event of the CNS.	Animal, in vitro, ex vivo and cellular studies; mixed populations without separately extractable MS data.
**Intervention (Concept)**	PUFA supplementation administered in clearly defined galenic dosage forms (capsules, oils or proprietary formulations), including omega-3, omega-6 and combined PUFA formulations.	Dietary patterns, dietary counselling, food consumption, naturally PUFA-rich foods, or studies assessing fatty acid status without supplementation.
**Combined interventions**	Multicomponent interventions in which the PUFA component was clearly identifiable, and its effects could be reasonably interpreted.	Multicomponent interventions where the PUFA-specific contribution could not be distinguished from that of other bioactive components.
**Context**	No restrictions regarding healthcare setting or geographical location.	
**Study designs**	Randomized controlled trials, non-randomized clinical trials, quasi-experimental studies, before-and-after studies, open-label intervention studies, prospective and retrospective cohort studies, and longitudinal cohort studies.	Cross-sectional studies, case-control studies, case reports and case series.
**Publication types**	Original full-text research articles and systematic reviews.	Narrative reviews, scoping reviews, umbrella reviews, editorials, letters, commentaries, conference abstracts (as standalone evidence), and protocols without results.
**Clinical trial registries**[1]	Registry records with publicly available extractable results or linked publications with extractable data.	Ongoing, recruiting, withdrawn, terminated, or completed trials without publicly available results.

Criterion	Inclusion criteria	Exclusion criteria
**Outcomes**	At least one clinical, imaging, laboratory/mechanistic, lipid-related or safety outcome related to PUFA supplementation.	No relevant outcome related to PUFA supplementation.
**Publication characteristics**	No restrictions on publication year or language.	

CNS: central nervous system; CIS: clinically isolated syndrome; MS: multiple sclerosis; PUFA: polyunsaturated fatty acid.
[1] Registry records without publicly available results were not included in the review.

Table S2. Electronic search strategies for each database and trial registry.

Information source	Search strategy
MEDLINE (via PubMed)	("multiple sclerosis"[tiab] OR RRMS[tiab] OR PPMS[tiab] OR SPMS[tiab]) AND ("polyunsaturated fatty acid*"[tiab] OR PUFA*[tiab] OR "omega-3"[tiab] OR "omega-6"[tiab] OR "n-3"[tiab] OR "n-6"[tiab] OR "fish oil"[tiab] OR "eicosapentaenoic acid"[tiab] OR "docosahexaenoic acid"[tiab]) NOT (animals[mh] NOT humans[mh])
Scopus	TITLE-ABS-KEY(("multiple sclerosis" OR RRMS OR PPMS OR SPMS) AND ("polyunsaturated fatty acid*" OR PUFA* OR "omega-3" OR "n-3 fatty acid*" OR "fish oil" OR "eicosapentaenoic acid" OR "docosahexaenoic acid"))
Web of Science Core Collection	("multiple sclerosis" AND ("polyunsaturated fatty acid*" OR PUFA* OR omega-3 OR omega-6 OR EPA OR DHA OR "fish oil"))
ScienceDirect	"multiple sclerosis" AND ("polyunsaturated fatty acids" OR PUFA OR PUFAs OR "omega-3" OR "omega-6" OR "fish oil")
CENTRAL	(multiple sclerosis):ti,ab,kw AND (PUFA):ti,ab,kw AND ("fatty acid"):ti,ab,kw

Information source	Search strategy
ClinicalTrials.gov	("multiple sclerosis" OR MS OR "clinically isolated syndrome" OR demyelination OR demyelinating) AND ("omega-3" OR "fish oil" OR EPA OR DHA)
	("multiple sclerosis" OR MS OR "clinically isolated syndrome" OR demyelination OR demyelinating) AND ("polyunsaturated fatty acid" OR PUFA)
	("multiple sclerosis" OR MS OR "clinically isolated syndrome" OR demyelination OR demyelinating) AND ("omega-6" OR "evening primrose oil" OR "hemp seed oil" OR GLA)
WHO ICTRP	multiple sclerosis AND (omega-3 OR omega 3 OR fish oil OR EPA OR DHA OR PUFA OR polyunsaturated fatty acid)
	multiple sclerosis AND (omega-6 OR omega 6 OR gamma-linolenic acid OR GLA)
EU CTR/EudraCT	Multiple sclerosis AND (fish oil OR PUFA OR polyunsaturated fatty acid OR EPA OR DHA OR omega-6 OR gamma-linolenic acid OR EPA OR DHA OR omega-6 OR gamma-linolenic acid)

Table S3. Structured data charting form and core variables

Domain	Variables charted
**Primary studies**	
Study identification	Evidence source, first author, year
Study characteristics	Study design
Population	Population studied, sample size

Domain	Variables charted
Intervention	PUFA formulation/exposure, comparator, intervention duration
Outcomes	Clinical outcomes, biological outcomes
Additional charting	Contextual methodological and outcome information relevant to the review objectives and evidence characterization; type of funding
**Systematic reviews**	
Review identification	Evidence source, first author, year
Review characteristics	Review type
Evidence base	Number of studies included
Intervention scope	PUFA interventions covered
Outcomes	Main outcomes
Additional charting	Search period covered, databases searched, critical appraisal or risk-of-bias assessment methods, certainty or overall quality of evidence (when reported), and contextual information relevant to the review objectives and narrative synthesis; type of funding

Table S4. Full-text articles excluded after eligibility assessment and reasons for exclusion.

Citation	Article title	Primary reason for exclusion
Abd-Elmawla, 2025	Role of vitamin D and omega 3 fatty acids in improving HDL biogenesis among multiple sclerosis patients via orchestrating CHROME/APOA1-AS/ABCA1/APOA1 milieu	Ineligible study design

Aupperle, 2008	Omega-3 fatty acids and multiple sclerosis: relationship to depression	Dietary or food-based exposure/intervention
Bjørnevik, 2017	Polyunsaturated fatty acids and the risk of multiple sclerosis	Dietary or food-based exposure/intervention
ClinicalTrials.gov (NCT00122954)	Fish Oil for the Treatment of Depression in Patients With Multiple Sclerosis	linked registry record represented by included publication
ClinicalTrials.gov (NCT00360906)	Omega-3 Fatty Acid Treatment in Multiple Sclerosis	linked registry record represented by included publication
ClinicalTrials.gov (NCT01381354)	Nutrition, Neuromuscular Electrical Stimulation (NMES) and Secondary Progressive Multiple Sclerosis (SPMS)	Ineligible publication type
ClinicalTrials.gov (NCT01842191)	Efficacy of fish oil on serum of TNF-α, IL-1β, and IL-6 oxidative stress markers in multiple sclerosis treated with interferon beta-1b	Linked registry record represented by included publication
ClinicalTrials.gov (NCT02939079)	Evaluating of the Effect of Fingolimod With Fish Oil on Relapsing-Remitting Multiple Sclerosis Patients	linked registry record represented by included publication
ClinicalTrials.gov (NCT05007483)	Efficacy of Diet on Quality of Life in Multiple Sclerosis	Dietary or food-based exposure/intervention
Daly, 2021	Omega-3 Index, fish consumption, use of fish oil supplements and first clinical diagnosis of central nervous system demyelination	Ineligible study design
EudraCT (2015-003147-19)	Phase III Neuroaspis PLP10 trial in relapsing multiple sclerosis	linked registry record represented by included publication
Field, 1977	Erythrocyte-UFA mobility test for pathogenesis and handling of the disease	Ineligible study design
Field, 1979	Polyunsaturated fatty acids and colchicine in multiple sclerosis	Ineligible publication type

Field, 1979	Polyunsaturated fatty acids in multiple sclerosis	Ineligible publication type
Gallai, 1992	Supplementation of polyunsaturated fatty acids in multiple sclerosis	Ineligible publication type
Guerrero, 2022	Efficacy of diet on fatigue, quality of life and disability status in multiple sclerosis patients: rapid review and meta-analysis of randomized controlled trials	Dietary or food-based exposure/intervention
Harbige, 1986	Dietary intervention studies on the phosphoglyceride fatty acids and electrophoretic mobility of erythrocytes in multiple sclerosis	Dietary or food-based exposure/intervention
Harbige, 2007	Polyunsaturated fatty acids in the pathogenesis and treatment of multiple sclerosis	Ineligible publication type
Holman, 1989	DEFICIENCIES OF POLY-UNSATURATED FATTY-ACIDS AND REPLACEMENT BY NONESSENTIAL FATTY-ACIDS IN PLASMA-LIPIDS IN MULTIPLE-SCLEROSIS	Ineligible study design
Horrobin, 1979	Multiple sclerosis: the rational basis for treatment with colchicine and evening primrose oil	Ineligible publication type
Kong-González, 2015	Polyunsaturated fatty acids for multiple sclerosis treatment: scientific evidence; [Ácidos grasos poliinsaturados en el tratamiento de la esclerosis multiple: evidencia científica]	Ineligible publication type
Lin, 2024	Diet and omega-3 and vitamin D supplement use predict five-year fatigue and disability trajectories in people with multiple sclerosis	Ineligible study design
Low, 2024	Vitamin, antioxidant and micronutrient supplementation and the risk of developing incident autoimmune diseases: a systematic review and meta-analysis	PUFA component or effect not separately interpretable
Marx, 2020	The effect of emerging nutraceutical interventions for clinical and biological outcomes in multiple sclerosis: a systematic review	No eligible PUFA supplementation
Mehta, 2009	Polyunsaturated fatty acids and their potential therapeutic role in multiple sclerosis	Ineligible publication type
Mertin, 1973	Unsaturated fatty acids in multiple sclerosis	Ineligible study design

Nordvik, 2000	Effect of dietary advice and n-3 supplementation in newly diagnosed MS patients	PUFA component or effect not separately interpretable
Petersen, 2021	A systematic review of possible interactions for herbal medicines and dietary supplements used concomitantly with disease-modifying or symptom-alleviating multiple sclerosis drugs	No relevant outcome
Rezapour-Firouzi, 2013	Association of Expanded Disability Status Scale and Cytokines after Intervention with Co-supplemented Hemp Seed, Evening Primrose Oils and Hot-natured Diet in Multiple Sclerosis Patients	PUFA component or effect not separately interpretable
Sasaki, 2015	Determination of ω-6 and ω-3 PUFA metabolites in human urine samples using UPLC/MS/MS	Absence of relevant data on multiple sclerosis
Solsona, 2024	Associations between diet and disease progression and symptomatology in multiple sclerosis: A systematic review of observational studies	Dietary or food-based exposure/intervention
Tredinnick, 2020	Evaluating the Effects of Dietary Interventions on Disease Progression and Symptoms of Adults with Multiple Sclerosis: An Umbrella Review	Ineligible study design
van Meeteren, 2005	Antioxidants and polyunsaturated fatty acids in multiple sclerosis	Ineligible publication type
Weinstock-Guttman, 2005	Low fat dietary intervention with omega-3 fatty acid supplementation in multiple sclerosis patients	PUFA component or effect not separately interpretable
Wergeland, 2012	Polyunsaturated fatty acids in multiple sclerosis therapy	Ineligible publication type
WHO ICTRP (ISRCTN06166891)	MINERAL study: Neuroaspis PLP10 as adjunctive treatment for relapsing multiple sclerosis	Linked registry record represented by included publication
WHO ICTRP (ISRCTN87818535)	PLP10 proof-of-concept trial in relapsing-remitting multiple sclerosis	Linked registry record represented by included publication

Wolters, 2021	Effects of n-3 polyunsaturated fatty acid supplementation in the prevention and treatment of depressive disorders— a systematic review and meta-analysis	Ineligible publication type

Note: This table lists reports excluded after full-text eligibility assessment based on the predefined eligibility criteria. Reports that could not be retrieved (n = 5) and duplicate bibliographic records identified during full-text assessment (n = 3) are not included.

Table S5. Evidence mapping interpretability

Mapping item	Categories
Reference	First author, year
Study design	Randomized clinical trial / Non-randomized intervention study / Cohort study
PUFA supplement description	Clear / Partially clear / Unclear
Dose and duration reporting	Both reported / Dose only / Duration only / Neither reported
Comparator	Independent comparator / Within-subject comparison (baseline) / None
Outcome domain(s)	Clinical / Imaging / Laboratory-immunological / Lipid-related / Safety / Multiple domains
PUFA-specific interpretability[1]	Yes / Partial / No
Rationale[2]	Short justification when the classification is not straightforward

[1] PUFA-specific interpretability reflects the extent to which the reported outcomes can reasonably be attributed to the defined PUFA intervention or PUFA formulation under investigation, irrespective of methodological quality or risk of bias.
[2] Rationale is provided only when the assigned category required clarification (e.g., partial intervention description or interpretability).

Table S6. Evidence mapping of study reporting

Reference[1]	Study design	PUFA supplement description	Dose and duration reporting	Comparator	Outcome domains	PUFA-specific interpretability	Rationale
**Omega-6 primary studies**							
Bates, 1977	Double-blind RCT	Clear	Both	Independent comparator	Clinical	Yes	NA.
Bates, 1978	Double-blind RCT	Clear	Both	Independent comparator	Multiple domains	Yes	NA
Field & Joyce, 1978	Longitudinal observational study	Clear	Both	Within-subject comparison (baseline)	Laboratory-immunological	Yes	No independent control group
Field & Joyce, 1983	Longitudinal observational study	Partially clear	Both	Within-subject comparison (baseline)	Laboratory-immunological	Yes	No independent control group; Naudicelle capsules were identified, but the complete PUFA composition was not reported.
**Omega-3 primary studies**							
Bates, 1989	Double-blind controlled trial	Clear	Both	Independent	Multiple domains	Yes	NA
Gallai, 1995	Non-randomized controlled trial	Clear	Both	Within-subject comparison (baseline)	Laboratory-immunological	Yes	Participants were compared longitudinally with their own baseline and with a healthy control group.
Golabi, 2025	Double-blind RCT	Clear	Both	Independent comparator	Multiple domains	Yes	NA

Majdinasab, 2026	Double-blind RCT	Clear	Both	Independent comparator	Laboratory-immunological	Yes	NA
Mohammed, 2026	Cohort study nested cross-sectional	Partially clear	Neither reported	Independent comparator	Multiple domains	Partial	Omega-3 administered as part of a multi-component pharmacological intervention, limiting attribution of effects to PUFA supplementation alone.
Ramirez-Ramirez, 2013	Double-blind RCT	Clear	Both	Independent comparator	Multiple domains	Yes	NA
Shinto, 2009	Open-label pilot study	Clear	Both	Within-subject comparison (baseline)	Multiple domains	Yes	Open-label study without an independent comparator.
Shinto, 2016	Double-blind RCT (pilot)	Clear	Both	Independent comparator	Multiple domains	Yes	NA
Sorto-Gomez, 2016	Double-blind RCT	Clear	Both	Independent comparator	Multiple domains	Yes	Reports additional outcome measures from the same clinical trial as Ramirez-Ramirez (2013).
Torkildsen, 2012	Double-blind RCT	Clear	Both	Independent comparator	Multiple domains	Yes	NA
Torres-Sanchez, 2018	Double-blind RCT	Clear	Both	Independent comparator	Multiple domains	Yes	Reports additional mechanistic outcomes from the same clinical trial as Ramirez-Ramirez (2013).

Zandi-Esfahan, 2017	Double-blind RCT	Clear	Both	Independent comparator	Multiple domains	Yes	Omega-3 supplementation evaluated as an adjunct to fingolimod therapy.
**Combined polyunsaturated fatty acid primary studies**							
Aristotelous, 2021	Double-blind RCT	Clear	Both	Independent comparator	Multiple domains	Yes	NA
Pantzaris, 2013	Double-blind RCT (phase II)	Clear	Both	Independent comparator	Multiple domains	Yes	NA
Pantzaris, 2022	Double-blind RCT (phase III)	Clear	Both	Independent comparator	Multiple domains	Yes	NA
Rezapour-Firouzi, 2013 (primary report) [37]	Double-blind RCT	Clear	Both	Independent comparator	Multiple domains	Partial	Multi-component intervention with concomitant dietary modification, limiting attribution of observed effects solely to the PUFA formulation.
Rezapour-Firouzi, 2013 (report) [34]	Double-blind RCT	Clear	Both	Independent comparator	Multiple domains	Partial	Reports additional outcomes from the same trial; interpretation remains limited by the multi-component intervention.
Rezapour-Firouzi, 2013 (report) [38]	Double-blind RCT	Clear	Both	Independent comparator	Multiple domains	Partial	Reports additional outcomes from the same trial; interpretation remains limited by the multi-component intervention.

Rezapour-Firouzi, 2014	Double-blind RCT	Clear	Both	Independent comparator	Multiple domains	Partial	Reports additional outcomes from the same trial; interpretation remains limited by the multi-component intervention.
Rezapour-Firouzi, 2015	Double-blind RCT	Clear	Both	Independent comparator	Multiple domains	Partial	Reports additional outcomes from the same trial; interpretation remains limited by the multi-component intervention.

NA: Not applicable

[1]Some publications report different outcome domains from the same underlying clinical trial and are presented separately because this evidence mapping characterizes reporting at the publication level rather than consolidating evidence sources.

Table S7. Methodological characteristics of the systematic reviews

**Review**	**Search period**	**Databases**	**Evidence synthesis**	**Risk of bias assessment**	**Reported evidence assessment[1]**
AlAmmar, 2021	2009–2018	PubMed, Oxford, Cochrane, Embase, International pharmaceutical abstract, PsychINFO, and clinical trials government	Qualitative synthesis	Cochrane RoB	Overall quality ≥75%
Bagur, 2017	Up to Sept 2015	PubMed and Scopus	Qualitative synthesis	Not reported	Not reported
Harirchian, 2022	1962–2019	PubMed, Web of Science, and EMBASE	Qualitative synthesis	Not reported	Not reported

Review	Search period	Databases	Evidence synthesis	Risk of bias assessment	Reported evidence assessment[1]
Hempel, 2017	Inception–Mar 2015	PubMed, EMBASE, AMED, SCOPUS, Web of Science, and GreenFILE	Qualitative synthesis	Cochrane RoB + GRADE	Moderate (dietary interventions including PUFAs) to very low (vitamin D)
Parks, 2020	Up to May 2019	PubMed, Embase, CENTRAL, Web of Science, ClinicalTrials.gov, and ICTRP	Meta-analysis + qualitative synthesis	Cochrane RoB	Most PUFA trials had unclear or high risk of bias in one or more domains
Sedighiyan, 2019	Up to Oct 2018	PubMed, SCOPUS, and Cochrane Library	Meta-analysis + qualitative synthesis	Cochrane RoB	Three trials rated as good quality; one as low quality

[1]Reported as presented by the original systematic reviews; different assessment methods were used and results are not directly comparable.

Table S8. Funding sources and institutional support reported by the included independent evidence sources.

Independent evidence source	Funding reported	Type of support
AlAmmar, 2021	No external funding reported.	None reported
Aristotelous, 2021	No external funding.	Two authors declared as inventors of the tested formulation
Bagur, 2017	European Regional Development Fund (FEDER).	Public research funding

Independent evidence source	Funding reported	Type of support
Bates, 1977	Multiple Sclerosis Society of Great Britain and Northern Ireland. Van den Berghs Ltd supplied dietary spreads.	Charity funding + study products supplied by industry
Bates, 1978	Multiple Sclerosis Society of Great Britain and Northern Ireland. Van den Berghs Ltd supplied dietary spreads.	Charity funding + study products supplied by industry
Bates, 1989	Multiple Sclerosis Society of Great Britain and Northern Ireland; John Harris Trust; Action MS–Northern Ireland; Dickinson Dees bequest. Marfleet Refining Co. supplied study capsules.	Charity funding + study products supplied by industry
Field & Joyce, 1978	Action for Research into Multiple Sclerosis (ARMS).	Charity funding
Field & Joyce, 1983	Action for Research into Multiple Sclerosis (ARMS).	Charity funding
Gallai, 1995	Carlo Erba Farmitalia supplied ESAPENT capsules.	Study products supplied by industry
Golabi, 2025	Vice-Chancellor for Research Affairs, Abadan University of Medical Sciences (Grant 1398T-716).	University/public research funding
Harirchian, 2022	Tehran University of Medical Sciences and Health Services.	University/public research funding
Hempel, 2017	U.S. Department of Veterans Affairs.	Government funding
Majdinasab, 2026	Funding not reported.	Not reported
Mohammed, 2026	No funding declared.	None

Independent evidence source	Funding reported	Type of support
Pantzaris, 2013	Cyprus Ministry of Commerce, Industry and Tourism (High-Technology Business Incubator Programme).	Government funding
Pantzaris, 2022	Cyprus Ministry of Energy, Commerce, Industry and Tourism (Structural Funds Programme 8.1.12.13.2.3.60).	Government funding
Parks, 2020	Funding not reported.	Not reported
Ramirez-Ramirez, 2013	Funding not reported.	Not reported
Rezapour-Firouzi (2013–2015)	Tabriz University of Medical Sciences (25%) and authors◎personal funding (75%).	University funding + personal funding
Sedighiyan, 2019	No funding declared.	None
Shinto, 2009	National Institutes of Health; U.S. Department of Veterans Affairs; Nancy Davis Center Without Walls. Vital Nutrients supplied study supplements.	Government/charitable funding + study products supplied by industry
Shinto, 2016	Nordic Naturals supplied study supplements and placebo capsules.	Study products supplied by industry
Torkildsen, 2012	Western Norway Regional Health Authority; Norwegian Multiple Sclerosis Society; Pronova Biocare; Amersham Health; Merck Serono.	Government and charity funding + industry support
Zandi-Esfahan, 2017	Vice-Chancellor for Research and Technology, Isfahan University of Medical Sciences (Grant 293396).	University/public research funding

Note: Funding information is reported as declared in the original publications. "Study products supplied by industry" indicates provision of supplements and/or placebo formulations and should not be interpreted as direct sponsorship unless explicitly stated by the original authors. "Funding not reported" indicates that no funding statement was identified in the publication.
