# Supplementary material for: Polyunsaturated Fatty Acid Supplementation in Multiple Sclerosis Management: A Scoping Review
Source: Nutrients. 2026 Sep 5;18(17):2915. doi: 10.3390/nu18172915 (PMC13567611; doi:10.3390/nu18172915)
Supplement: Supplementary file 1 [file nutrients-18-02915-s001.zip › Supplementary File S1_revised_26 08 26.pdf]

Supplementary File S1. Operational framework for AI-assisted screening and data charting

S1.1 AI tool configuration

**Item**	**Description**	
LLM provider	OpenAI	Google
Interface	ChatGPT	NotebookLM
Model	GPT-5.5 Thinking	Gemini model via NotebookLM; specific underlying model version not displayed in the user interface
Date(s) of use	Title and abstract screening: 12–13 June 2026; other uses during manuscript preparation: June–July 2026 (individual access dates not systematically recorded).	Data charting: 3–6 July 2026; initial infographic generation for Figure 2: 22 July 2026
Purpose	Initial title and abstract screening; language and textual coherence optimization during manuscript preparation.	Data charting support from uploaded full-text articles; generation of the initial infographic used for Figure 2.
Input	Database search-result files containing titles, abstracts where available, and bibliographic metadata, together with the predefined operational screening criteria; author-provided manuscript text for language and textual coherence optimization	Uploaded full-text publications together with standardized data-charting instructions; author-provided content and instructions for the initial infographic used for Figure 2.
Output	Record-level Include, Uncertain, or Exclude classification with a brief justification; identification of potential bibliographic duplicates; language and textual editing suggestions.	Structured retrieval and organization of predefined study characteristics and outcome data; initial infographic used as the basis for Figure 2.

Item	Description	
Output handling	All potential duplicates and Exclude classifications were manually verified; AI classifications were not used as final exclusion decisions without human verification	All retrieved information used in the review was checked against the corresponding original publication before inclusion; the initial infographic was subsequently reviewed and refined by the authors for scientific accuracy.
Human oversight	Human verification of all excluded records and bibliographic duplicates; all *Include* and *Uncertain* records advanced to full-text screening	Human verification of all retrieved information against the original publications before inclusion; uncertain or ambiguous cases discussed with a second reviewer
Sampling parameters	Default ChatGPT settings (not user configurable)	Default NotebookLM settings (not user-configurable)
Fine tuning	None	None
External retrieval	None	None (operated exclusively on uploaded full-text articles)

S1.2 Operational screening framework

Framework	PCC: Population = people with MS/CIS/first demyelinating event; Concept = PUFA supplementation in galenic/pharmaceutical/nutraceutical dosage forms; Context = human experimental, cohort, and systematic review evidence.
Included evidence sources	Randomized clinical trials; non-randomized clinical trials; quasi-experimental/open-label/before-after studies; prospective/retrospective cohort studies of PUFA supplement use; systematic reviews of eligible studies.
Supplement concept	PUFAs, omega-3, omega-6, EPA, DHA, ALA, GLA, fish oil supplement, evening primrose oil supplement, hemp seed oil supplement, and clearly defined PUFA-containing formulations.

Not included as concept	Foods or diets rich in PUFAs, fish/salmon consumption, dietary patterns, broad dietary counselling, lipid biomarkers without supplement use.
Outcomes	Clinical, imaging, laboratory/immunological, lipid-related, and safety outcomes. Lipid profile is treated only as an outcome.
Screening approach	Automated operational title/abstract screening using the refined protocol. Records marked "Uncertain" require full-text/manual confirmation.
Deduplication	Primary key DOI when available; otherwise normalized title. Source-level decisions were retained in the detailed sheets.

S1.3 Classification categories

Classification	Operational definition
Include	Title/abstract clearly fulfilled the predefined eligibility criteria.
Uncertain	Available information was insufficient to determine eligibility; record forwarded to full-text assessment.
Exclude	Title/abstract clearly failed one or more eligibility criteria.

S1.4 Deduplication procedure

Bibliographic duplicates were identified using DOI as the primary identifier and normalized titles when DOI was unavailable. Duplicate classifications generated by the LLM were verified by the human reviewer before record removal.

S1.5 Human verification workflow

Stage	Performed by	Output	Human oversight
Bibliographic import	Human	Database exports	—
Deduplication	LLM	Duplicate records identified	All duplicates verified
Title/abstract screening	LLM	Include / Uncertain / Exclude + justification	All exclusions verified[1]

Stage	Performed by	Output	Human oversight
Full-text assessment	Two reviewers	Final inclusion/exclusion	Consensus; third reviewer if required
Data charting	NotebookLM + one reviewer	Retrieval of predefined data items + contextual information from included full-text articles	All retrieved information was verified against the original publications; uncertain or ambiguous case discussed with a second reviewer.

[1]All 1,960 records classified as Exclude by the LLM underwent human verification. Of these, 1,959 (99.95%) were confirmed as exclusions and one record (0.05%) was reclassified for full-text assessment.

S1.6 ChatGPT screening prompt

Context	Title / abstract screening was conducted in ChatGPT on 12–13 June 2026. For each database, the corresponding search-result file was provided to ChatGPT. The screening instruction was given in Portuguese and referred to the operational criteria defined in the preceding interaction and reproduced in Section S1.2. Because the screening prompt was context-dependent, the textual prompt and the operational criteria to which it referred should be considered together when reproducing the screening procedure.
Original prompt (Portuguese, textually)	"Aplica os critérios operacionais para triagem de título e resumo aos resultados que obtive."
English translation	"Apply the operational criteria for title and abstract screening to the results obtained."

S1.7 NotebookLM data-charting prompts

Context	NotebookLM-assisted data charting was conducted on 3–6 July 2026 using standardized prompts tailored to the type of evidence source. For primary studies, the same prompt structure was applied across the predefined PUFA intervention groups, with adaptations.
Original prompt for	Create a table titled "Characteristics of included systematic reviews" using only the information available in the uploaded sources.

systematic reviews	Use the following columns: Author, Year; Review type; Studies included; PUFA interventions covered; Main outcomes<br><br>For each included systematic review, extract: first author surname and publication year; type of review, such as systematic review, meta-analysis, narrative review, scoping review, or other, as stated by the authors; number and type of studies included, if reported; PUFA interventions covered, specifying omega-3, omega-6, combined PUFAs, EPA, DHA, GLA, linoleic acid, alpha-linolenic acid, oils, or specific formulations when available; outcomes assessed and results, including clinical, relapse-related, disability, inflammatory, MRI, safety, quality of life, biochemical, or other outcomes.<br><br>If information is not reported or cannot be clearly extracted from the source, write "Not reported". Do not infer information beyond the source text. Keep the wording concise and suitable for direct use in a manuscript table.
Original prompt for primary studies	Create a table titled "Characteristics of included [PUFA] primary studies" using only the information available in the uploaded sources.<br><br>Use the following columns:<br><br>Author, Year ǀ Design ǀ Population ǀ Intervention/exposure ǀ Comparator ǀ Duration ǀ Clinical outcomes ǀ Biological outcomes<br><br>For each included study, extract: first author surname and publication year; Design type of study, such as RCT, prospective observational or other, as stated by the authors; Population as phenotype of MS; PUFA interventions covered, specifying omega-3, omega-6, combined PUFAs, EPA, DHA, GLA, linoleic acid, alpha-linolenic acid, oils, or specific formulations when available; outcomes assessed and results, including clinical, relapse-related, disability, inflammatory, MRI, safety, quality of life, biochemical, or other outcomes.<br><br>If information is not reported or cannot be clearly extracted from the source, write "Not reported". Do not infer information beyond the source text. Keep the wording concise and suitable for direct use in a manuscript table.
